# CDK5-mediated phosphorylation of XBP1s contributes to its nuclear translocation and activation in MPP^+^-induced Parkinson’s disease model

**DOI:** 10.1038/s41598-017-06012-6

**Published:** 2017-07-17

**Authors:** Feng-Juan Jiao, Qing-Zhi Wang, Pei Zhang, Jian-Guo Yan, Zheng Zhang, Feng He, Qian Zhang, Ze-Xi Lv, Xiang Peng, Hong-Wei Cai, Bo Tian

**Affiliations:** 1 Department of Neurobiology, Tongji Medical School, Huazhong University of Science and Technology, 13 Hangkong Road, Wuhan, Hubei Province 430030 P. R. China; 2 Institute for Brain Research, Huazhong University of Science and Technology, 13 Hangkong Road, Wuhan, Hubei Province 430030 P. R. China; 3 Medical School, Hubei Ploytecnic University, Huangshi, Hubei Province P. R. China

## Abstract

Parkinson’s disease (PD) is an irreversible and progressive neurodegenerative disorder characterized by the selective loss of dopaminergic neurons of the substantia nigra pars compacta. Growing evidence indicates that endoplasmic reticulum stress is a hallmark of PD; however, its exact contribution to the disease process remains poorly understood. Here, we used molecular biology methods and RNA-Seq analysis to explored an unexpected role of spliced X-Box binding protein 1 (XBP1s) in the nervous system. In this study, we determined that the IRE1α/XBP1 pathway is activated in MPP^+^-treated neurons. Furthermore, XBP1s was identified as a substrate of CDK5 and that the phosphorylation of XBP1s at the Ser61 residue enhances its nuclear migration, whereas mutation of the residue to alanine substantially reduces its nuclear translocation and activity. Importantly, phosphorylated XBP1s acts as a nuclear transcription factor for multiple target genes, including metabolic-related genes, FosB, and non-coding RNAs. Our findings confirm that the IRE1α/XBP1 pathway is activated in PD, and reveal a novel role of XBP1s in the pathogenesis of PD. This pathway may be a new therapeutic strategy for PD.

## Introduction

Parkinson’s disease (PD) is a progressive neurodegenerative disease characterized by specific motor symptoms such as resting tremor, rigidity, bradykinesia, hypokinesia, and akinesia, affecting 1% of the population over 60 years of age^1^. The pathological hallmarks of PD are loss of dopaminergic neurons in the substantia nigra pars compacta and the formation of Lewy bodies^2^. Several perturbations in cellular homeostasis occur in PD, including protein maturation, calcium homeostasis, oxidative stress, mitochondrial dysfunction, and proteasome function^3, 4^. Accumulating evidence in genetic and toxicological models of PD suggests that the disruption of the secretory pathway leads to pathological levels of endoplasmic reticulum (ER) stress^5–7^. Early-symptomatic animals overexpressing α-synuclein showed α-synuclein oligomers in the ER lumen and ER stress^8^. Additional evidence for the involvement of ER stress in PD was provided by studies with neurotoxins, including 6-hydroxydopamine (6-OHDA), rotenone and N-methyl-4-phenyl-1,2,3,6-tetrahydropyridine (MPTP) or its active derivative MPP^+^. These toxins are used to mimic the disease process both *in vitro* and *in vivo* ^9–11^.

Cells adapt to ER stress by activating a group of complex signal-transduction pathways, called the unfolded protein response (UPR). The UPR is initiated by the activation of three stress sensors located at the ER membrane: PRKR-like ER kinase, activating transcription factor 6, and inositol-requiring kinase 1α (IRE1α)^12^. The most conserved UPR signaling pathway is initiated by the activation of IRE1α, which is a serine-threonine kinase and endoribonuclease. The endoribonuclease activity of IRE1α cleaves XBP1 mRNA to initiate the removal of a 26-basepair intron, leading to a frameshift and the generation of an active b-ZIP transcription factorXBP1s, which contains a C-terminal transactivation domain absent from the unspliced form XBP1u^13^. Studies in animal models revealed that XBP1s is important for liver lipogenesis, inflammation, and energy metabolism^14^. A study using cell culture and a PD mouse model reported that XBP1s overexpression was cytoprotective against MPP^+^ and MPTP-induced cell death^15^. However, the mechanism of the IRE1α/XBP1 pathway in PD remains unknown.

In our present study, we identified a new pattern of phosphorylation-based regulation of XBP1s by cyclin-dependent kinase 5 (CDK5), a Ser/Thr kinase regulated by activator p35 or p39 but not the cell cycle^16^. Growing evidence indicates that CDK5 dysregulation is involved in the pathogenesis of neurodegenerative diseases such as Alzheimer’s disease and PD^17–19^. Our discovery of the relationship between CDK5 and XBP1s enhances our understanding of the role of the IRE1α/XBP1 pathway in neurons and highlights the regulatory mechanism of XBP1s in the pathogenesis of PD.

## Results

### Nuclear translocation of XBP1s is CDK5-dependent in MPP^+^-induced Parkinson’s disease model

**Figure 1:**
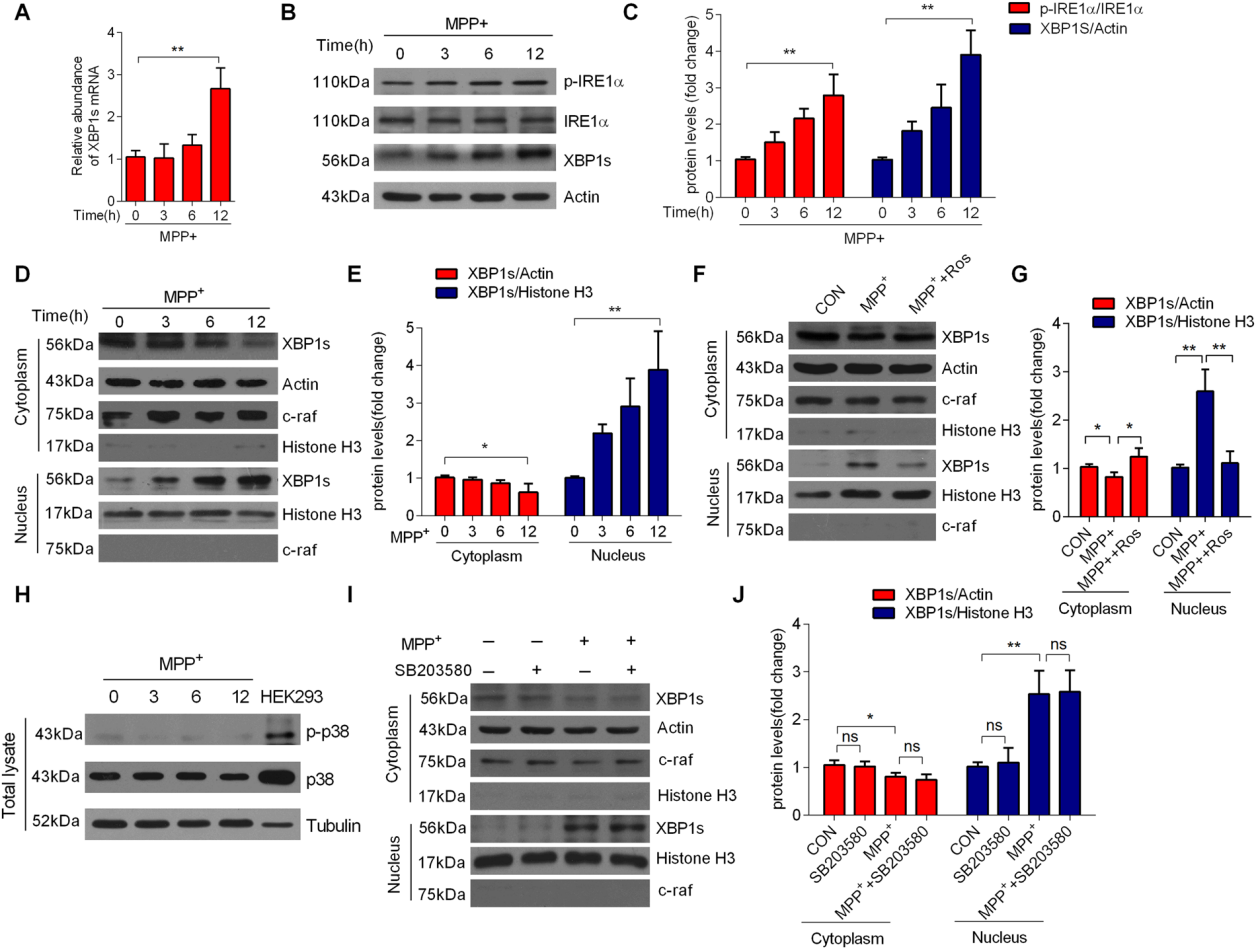
Nuclear translocation of XBP1s occurs in a CDK5-dependent manner in MPP^+^-induced Parkinson’s disease model (**A**) A longer treatment with MPP^+^ (100 μM) upregulated XBP1s messenger RNA (mRNA) in primary cultured cortical neurons. (**B**) MPP^+^-induced increases in the levels of phospho-Ser724-IRE1α (p-IRE1α), IRE1α, and XBP1s in cortical neurons. (**C**) Relative levels of p-IRE1α and XBP1s were quantified by densitometry. (**D**) Cytoplasmic and nuclear XBP1s protein levels from primary cultured cortical neurons treated with MPP^+^. (**E**) The relative level of XBP1s protein in cytoplasmic and nuclear lysates of MPP^+^-treated neurons were quantified by densitometry. (**F**) Pretreatment with Roscovitine (Ros) (10 μM) reduced the MPP^+^-induced nuclear translocation of XBP1s in neurons. Primary cultured neurons were pretreated with Ros (10 μM, 30 min), and then incubated the neurons with MPP^+^ (100 μM, 12 h). (**G**) The relative level of XBP1s protein in cytoplasmic and nuclear lysates of Ros and MPP^+^-treated neurons was quantified by densitometry. (**H**) Expressions of p-p38and p38 in the total lysates of MPP^+^-treated neurons. The total lysates of HEK293 cells was used as a standard control, and tubulin was used as a loading control. (**I**) Pretreatment with SB203580 (10 μM) had no effect on the nuclear translocation of XBP1s in MPP^+^-treated neurons. (**J**) The relative level of XBP1s protein in cytoplasmic and nuclear lysates of SB203580 and MPP^+^-treated neurons was quantified by densitometry. Ros is an inhibitor of CDK5 and SB203580 is an inhibitor of p38 MAPK. The membranes were re-probed for c-raf and Actin as markers and loading controls of cytoplasmic fractions respectively, and Histone H3 as markers and loading controls of nuclear fractions. Data are mean ± s.d. of n = 3 independent experiments. Significance was determined by unpaired Student’s t test (**A**,**C**,**E**,**G** and **J**). *P < 0.05, **P < 0.01.

Previous studies showed that the UPR is activated in neurodegenerative diseases. Upon UPR activation, XBP1 mRNA is cleaved by the activation of IRE1α, which subsequently generates XBP1s, a marker of the UPR^20^. XBP1s mRNA was significantly increased after 6 h and peaked after approximately 12 h (Fig. 1A) in primary cortical neurons exposed to MPP^+^^21^. Consistently, XBP1s protein and phospho-IRE1α (pIRE1α) levels peaked after a 12 h exposure (Fig. 1B and C).

XBP1s is an active transcription factor that translocates to the nucleus and regulates a subset of UPR target genes. We assessed the nuclear import of XBP1s in MPP^+^-treated cortical neurons. Cortical neurons incubated with MPP^+^ (100 μM) showed a higher translocation of XBP1s to the nucleus over time (Fig. 1D and E). CDK5 activation was previously shown in many PD cell cultures and animal models. Thus, we investigated if the increased nuclear import of XBP1s was mediated by CDK5. We pretreated cortical neurons with Roscovitine (Ros) (10 μM, 30 min), a chemical inhibitor of CDK5 kinase, and then incubated the neurons with MPP^+^ (100 μM) for 12 h. Ros markedly reduced the nuclear import of XBP1s protein induced by MPP^+^ (Fig. 1F and G, Supplementary Fig. S1A and B).Closely correlated with this, inhibition of CDK5 RNAi lentivirus, reduced MPP^+^-induced nuclear import of XBP1s (Supplementary Fig. S1C and D), indicating that CDK5-mediated the nuclear translocation of XBP1s precedes. Moreover, primary cultured neurons treated with rotenone, another neurotoxicity in dopaminergic neurons for experimental models of PD^22^, similarly showed a higher translocation of XBP1s to the nucleus (Supplementary Fig. S2A and B), and the nuclear translocation of XBP1s reduced after pretreating with Ros (Supplementary Fig. S2C and D). In addition, we observed that inhibition of CDK5 by Ros did not affect the expression levels of pIRE1α in cortical neurons treated with MPP^+^ (Supplementary Fig. S3A and B).

XBP1s is phosphorylated by p38 mitogen-activated protein kinase (p38 MAPK) in severely obese and diabetic mice^23^. We failed to observe p38 MAPK expression at any time during the incubation of cortical neurons with MPP^+^ (Fig. 1H). Moreover, inhibition of p38 MAPK by SB203580, a chemical inhibitor of p38 MAPK, did not affect the nuclear import of XBP1s proteins (Fig. 1I and J). Our data showed that the IRE1α/XBP1 pathway was activated, which was independent on CDK5, and the increased translocation of XBP1s was dependent on CDK5 but not p38 MAPK in MPP^+^-treated neurons.

### CDK5 mediates the phosphorylation of XBP1s

**Figure 2:**
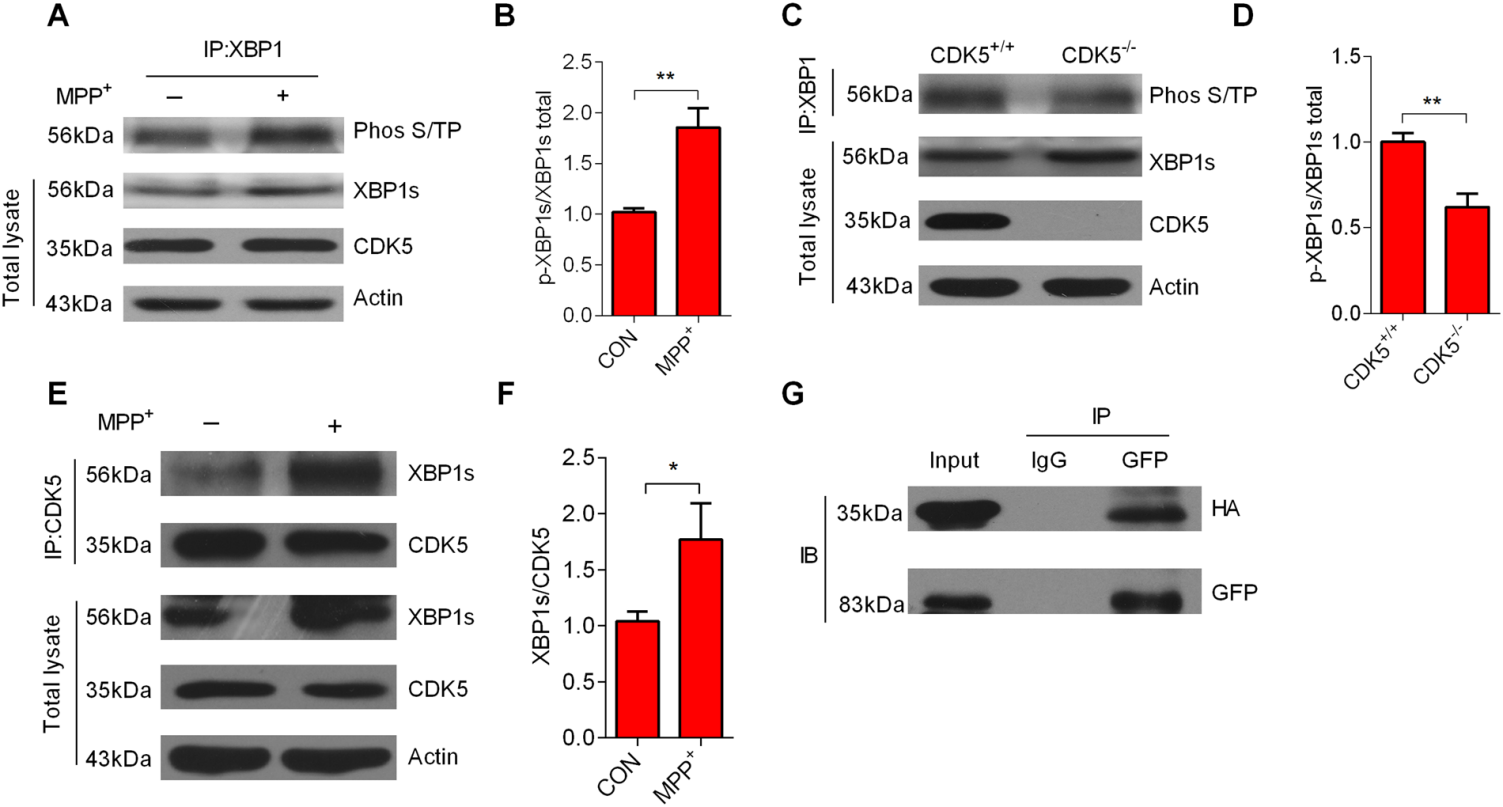
CDK5 mediates the phosphorylation of XBP1s. (**A**) The expression level of phospho-XBP1s (p-XBP1s) was increased in neurons treated with MPP^+^(100 μM) for 12 h. Immunoprecipitation was performed using an XBP1 antibody with neuron total lysates, and then samples were analyzed by immunoblotting using a Phos S/T antibody. (**B**) The relative level of p-XBP1s protein in neurons was quantified by densitometry. (**C**) CDK5 phosphorylated XBP1s in *CDK5* ^+/+^ but not *CDK5* ^−/−^ mouse brain lysates. Immunoprecipitation was performed using an XBP1 antibody with cortical tissue lysates from CDK5 knockout mice, and then samples were analyzed by western blotting using a Phos S/T antibody. (**D**) The relative level of p-XBP1s protein in *CDK5* ^+/+^ and *CDK5* ^−/−^ mouse brain lysates was quantified by densitometry. (**E**) The interaction between XBP1s and CDK5 was increased in MPP^+^-treated neuron total lysates. Immunoprecipitation was performed using a CDK5 antibody with or without MPP^+^-treated neuron extracts, and then samples were analyzed by immunoblotting using an XBP1 antibody. (**F**) The graph is the quantification of the co-immunoprecipitations. (**G**) XBP1s co-immunoprecipitated with CDK5 in HEK293 cell lysates that co-expressed XBP1s and CDK5. HEK293 cells were co-transfected with HA-CDK5 and GFP-XBP1s WT plasmids, and then collected and analyzed 24 h after transfection. Subsequently, CDK5 was immunoprecipitated with a GFP antibody, and the precipitate was blotted with an HA antibody specific for CDK5. Data are mean ± s.d. of n = 3 independent experiments. Significance was determined by unpaired Student’s t test (B, D and F). *P < 0.05, **P < 0.01.

We investigated if XBP1s phosphorylation by CDK5 enhanced its nuclear migration. Indeed, the phosphorylation of XBP1s was significantly increased in MPP^+^-treated neurons when compared with controls (Fig. 2A and B). Furthermore, we showed that the levels of phosphorylated XBP1s were reduced in the brains of CDK5^−/−^ mice (Fig. 2C and D). The above findings indicate that CDK5 plays a role in XBP1s’ nuclear migration. To further investigate the endogenous interaction between XBP1s and CDK5, we completed an immunoprecipitation in neuron with or without MPP^+^ treatment (12 h) with control IgG and CDK5 antibodies. The complexes were analyzed using Western blots with an XBP1 antibody. Immunoprecipitation with the CDK5 antibody showed an increased XBP1s signal in MPP^+^-treated neurons compared with control group (Fig. 2E and F). To confirm the interaction between XBP1s and CDK5 *in vitro*, we overexpressed mouse XBP1s and CDK5 by transfecting HEK293 cells with GFP-tagged XBP1s and HA-tagged CDK5 plasmids. Immunoprecipitation of CDK5 using a GFP antibody, followed by western blotting with an HA antibody specific for CDK5, revealed that CDK5 immunoprecipitates with XBP1s (Fig. 2G). Taken together, these results indicate that CDK5 interacts directly with XBP1s, and the binding of XBP1s to CDK5 increased in MPP^+^-treated neurons.

### CDK5 phosphorylates XBP1s at residue Ser61

**Figure 3:**
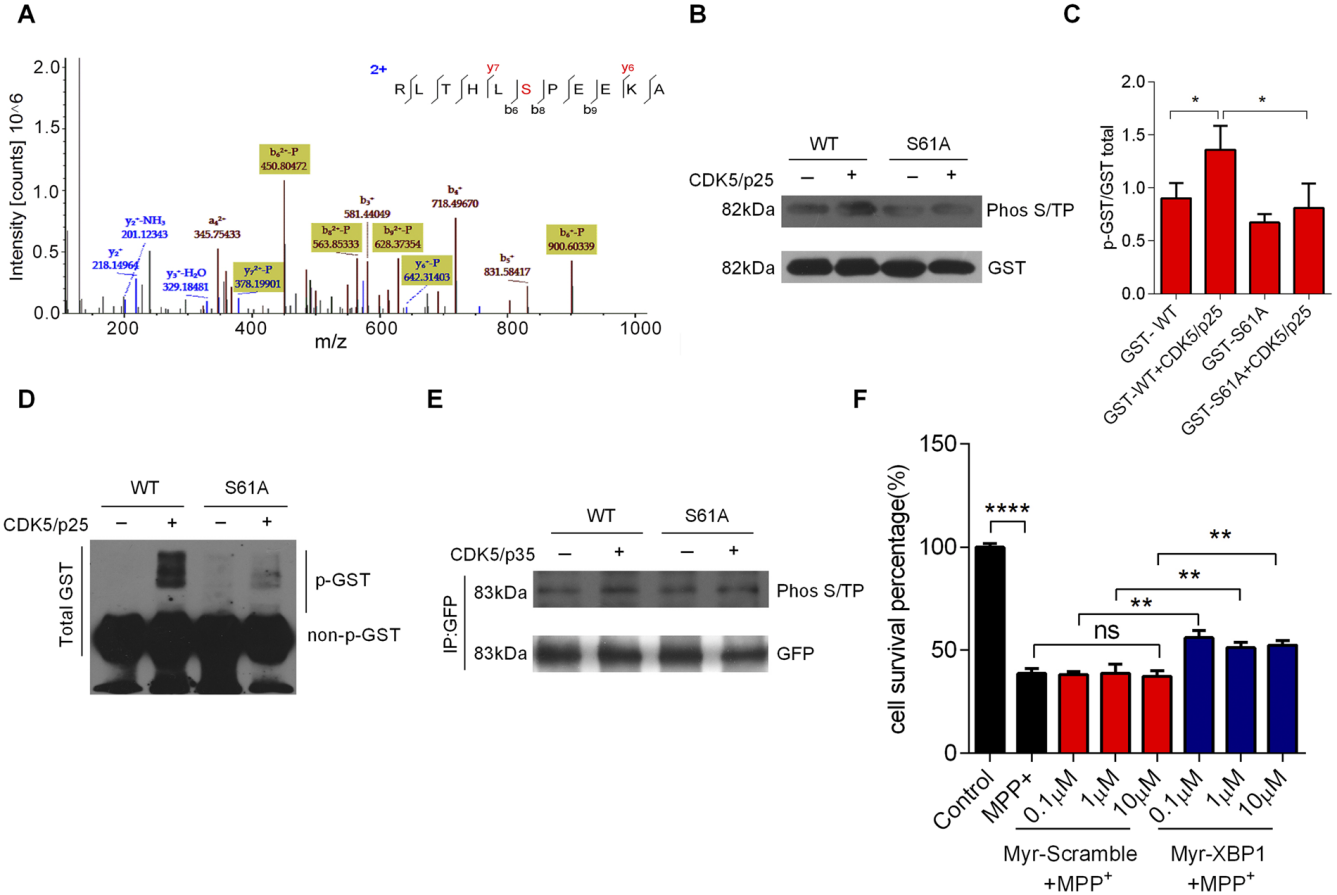
CDK5 phosphorylates XBP1s at Ser61. (**A**) Mass spectrometry showed that CDK5 phosphorylated XBP1s at Ser61. The peptide RLTHLSPEEKA, which contained the XBP1s Ser61 residue, was synthesized *in vitro*. (**B**) CDK5/p25 phosphorylated XBP1s at Ser61 in an *in vitro* kinase assay. Purified glutathione-S-transferase (GST)-XBP1s WT or S61A fusion proteins were mixed with active CDK5/p25, and the phosphorylation signal was analyzed using a Phos S/T antibody. (**C**) Statistical analysis of the expression of phosphorylated GST-XBP1s WT or S61A fusion proteins. (**D**) The phosphorylation signal of GST-XBP1s WT or S61A fusion proteins was analyzed by Phos-tag SDS-PAGE gels**. (E)** CDK5/p35 phosphorylated XBP1s at Ser61 in HEK293 cells. HEK293 cells were co-transfected with HA-CDK5/Myc-p35 and GFP-XBP1s-WT or GFP-XBP1s-S61A plasmids, and then collected and immunoprecipitated with a Phos S/T antibody 24 h after transfection. (**F**) The transmembrane peptide Myr-XBP1, which contained the XBP1s Ser61 residue, had a protective effect on MPP^+^-induced cell death. Primary cultured neurons were pretreated with XBP1 peptide or scrambled peptide (0.1 μM, 1 μM, 10 μM, 30 min), and then treated with MPP^+^ (250 μM, 24 h). Cell survival was visualized by MTT assay. Data are mean ± s.d. of n = 3 independent experiments. Significance was determined by unpaired Student’s t test (**C** and **F**). *P < 0.05, **P < 0.01, ****P < 0.0001.

To verify a kinase–substrate relationship, we analyzed the protein sequence of XBP1s with two bioinformatic tools: GPS^24^ and Scansite^25^ (http://scansite.mit.edu/). We identified a candidate site at the serine residue at position 61 (Ser61), which was highly conserved across sequences of all available species in the examined databases. Next, we synthesized an 11-amino acid peptide containing XBP1s Ser61 (sequence RLTHLSPEEKA), and then incubated the peptide with CDK5/p25 kinase *in vitro* for 30min. Tandem mass spectrometry showed that CDK5 directly phosphorylated XBP1s at Ser61 (Fig. 3A). We designed a mutant of XBP1s in which Ser61 was mutated to alanine (S61A). The CDK5/p25 complex phosphorylated wild-type (WT) XBP1s but not the S61A mutant in a kinase assay (Fig. 3B, C and D). To confirm this result, immunoprecipitation experiments using HEK293 cells co-transfected with HA-tagged CDK5/p35, GFP-tagged WT XBP1s, or GFP-tagged S61A mutant plasmids were conducted. These data showed that the CDK5/p35 complex phosphorylated WT XBP1s but not the S61A mutant (Fig. 3E). These observations indicate that CDK5 directly phosphorylates XBP1s at Ser61 site.

To clarify the role of this phosphorylation site, we synthesized a scrambled peptide and a myristic acid (Myr)-modified peptide containing the XBP1s Ser61 site (Myr-XBP1) that could enter neurons and competitively bind with CDK5 to block the phosphorylation of XBP1s at Ser61. Alexander *et al*. have reported that using peptide modified with myristic acid in its N terminus could induce an efficient penetration into cells^26, 27^. We pretreated cortical neurons with the transmembrane peptide for 30min, and then incubated neurons with MPP^+^ for 24h. A MTT analysis showed that the peptides have no effect on the cell survival without MPP^+^ (Supplementary Fig. S4A), and Myr-XBP1 peptide had obviously protective effects on MPP^+^-induced neuronal death compared with scrambled peptide control (Fig. 3E). Moreover, our findings also revealed that the expression of cleaved caspase-3 was increased in MPP^+^-treated neurons, and Myr-XBP1 peptides had protective effect on MPP^+^-induced cell apoptosis (new Supplementary Fig. S4B and C). Thus, our results indicate that the phosphorylation of XBP1s at Ser61 by CDK5 mediates neuronal death.

### Phosphorylation of XBP1s enhances its nuclear translocation

**Figure 4:**
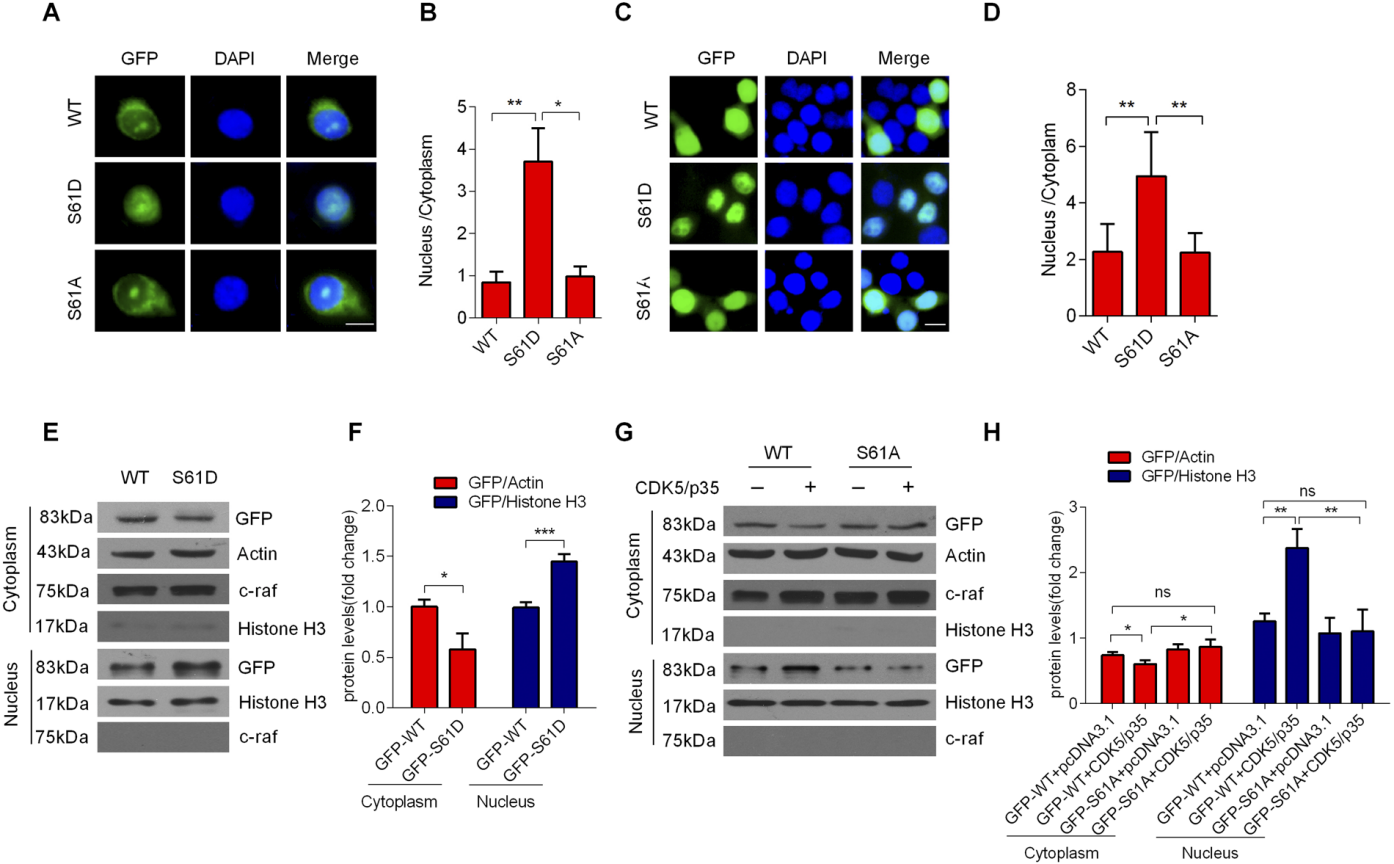
Phosphorylation of XBP1s at Ser61 facilitates its nuclear translocation. Mutation of Ser61 to Asp (S61D) enhanced the nuclear translocation of XBP1s in neurons (**A**) and HEK293 cells (**C**) transiently transfected with GFP-XBP1s-WT, GFP-XBP1s-S61A mutation or GFP-XBP1s-S61D mutation. Statistical analysis of the fluorescent intensity of nucleus/cytoplasm in neurons (**B**) and HEK293 cells (**D**) transfected with GFP-XBP1s-WT, GFP-XBP1s-S61A mutation or GFP-XBP1s-S61D mutation. (**E**) Mutation of S61D enhanced the nuclear translocation of XBP1s. The expression levels of GFP-XBP1s protein in cytoplasmic and nuclear lysates from HEK293 cells transfected with GFP-XBP1s-WT or GFP-XBP1s-S61D were detected by immunoblotting. (**F**) Statistical analysis of the expression of GST-XBP1s WT or S61D proteins in cytoplasmic and nuclear lysates from HEK293 cells transfected with GFP-XBP1s-WT or GFP-XBP1s-S61D plasmids. (**G**) CDK5/p35 enhanced the nuclear translocation of GFP-XBP1s-WT but not GFP-XBP1s-S61A in HEK293 cells. HEK293 cells were co-transfected with HA-CDK5/Myc-p35 and GFP-XBP1s-WT or GFP-XBP1s-S61A plasmids, and then collected and analyzed 24 h after transfection. (**H**) Statistical analysis of the expression of GST-XBP1s WT or S61A proteins in cytoplasmic and nuclear lysates from HEK293 cells co-transfected with HA-CDK5/Myc-p35 and GFP-XBP1s-WT or GFP-XBP1s-S61A. The membranes were re-probed for c-raf and Actin as markers and loading controls of cytoplasmic fractions respectively, and Histone H3 as markers and loading controls of nuclear fractions. The scale bar represents 10 μm. Data are mean ± s.d. of n = 3 independent experiments. Significance was determined by unpaired Student’st test (**B**,**D**,**F** and **H**). *P < 0.05, **P < 0.01, ***P < 0.001.

We created a mutant XBP1s by converting Ser61 to aspartic acid (S61D). Interestingly, overexpression of the GFP-tagged WT XBP1s, GFP-tagged S6A mutant XBP1s or GFP-tagged phosphomimetic mutant of XBP1s (S61D) in neurons (Fig. 4A and B) and HEK293 cells (Fig. 4C and D), respectively, showed that the GFP-tagged WT XBP1s and GFP-tagged S6A mutant XBP1s protein were expressed in the cytoplasm and nucleus, whereas the phosphomimetic mutant S61D protein was mainly localized in the nucleus. Western blots indicated that mutant S61D overexpression enhanced the transport of XBP1s to the nucleus (Fig. 4E and F). To exclude a possible overlapping function for the regulation of the nuclear translocation of XBP1s, we overexpressed the CDK5/p35 complex with the WT or S61A mutant XBP1s in HEK293 cells. After 24 h, we assessed the expression of XBP1s proteins in nuclear extracts. The nuclear translocation of WT XBP1s was significantly increased with the overexpression of CDK5/p35 complex, whereas the Ser61 mutant showed decreased nuclear translocation when compared with the WT XBP1s (Fig. 4G and H). These findings indicate that the phosphorylation of Ser61 is crucial for the nuclear translocation of XBP1s.

### Phosphorylated XBP1s acts as a transcription factor of multiple genes

**Figure 5:**
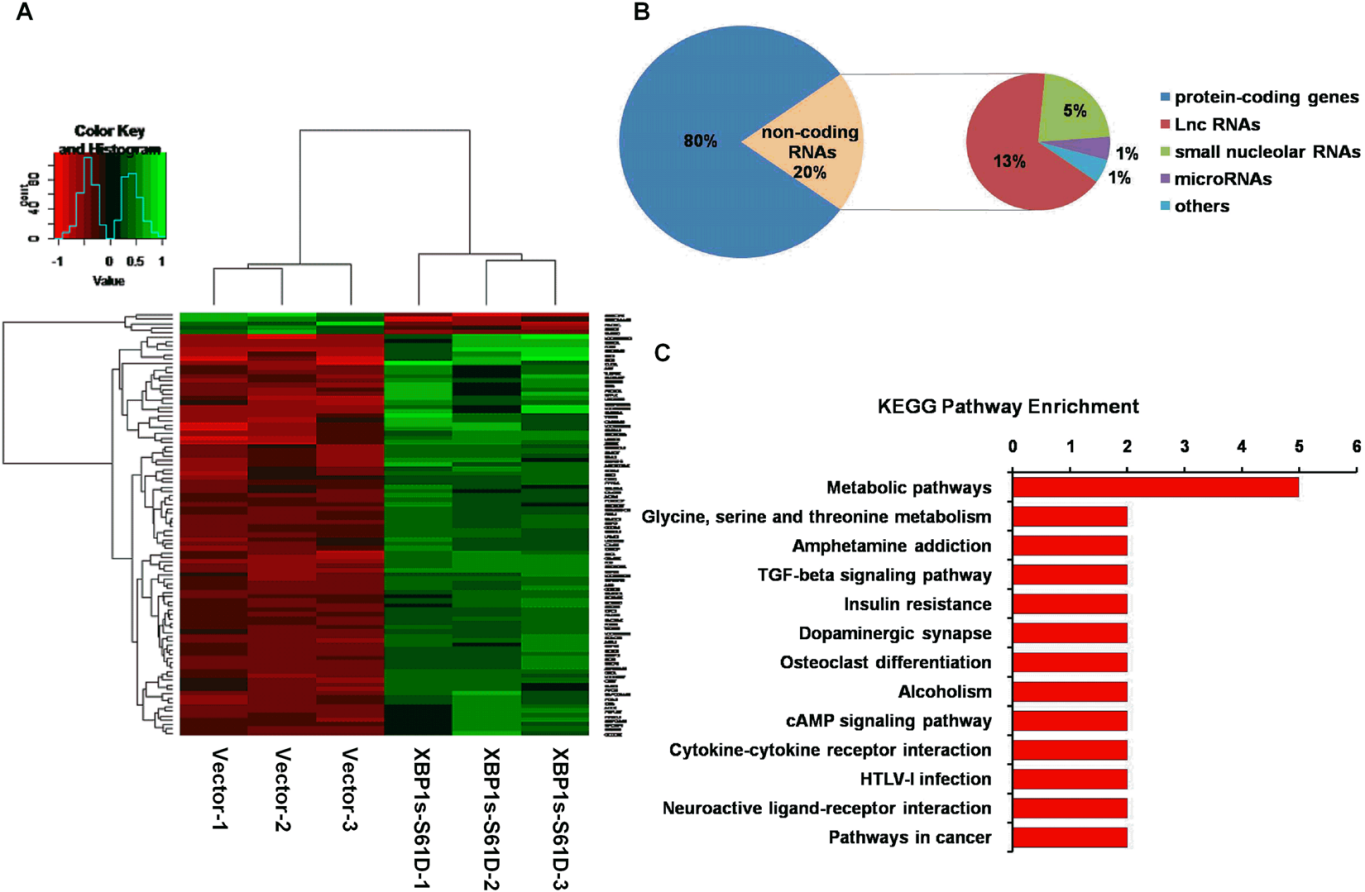
Phosphorylated XBP1s acts as a transcription factor to induce the expression of multiple genes. (**A**) Unsupervised hierarchical clustering analysis of the differentially expressed genes between overexpressing vectors and XBP1s-S61D plasmid cell groups (based on fold change ≥2, *P* < 0.01). The rows represent different genes, and the columns represent overexpressing vectors and XBP1s-S61D samples. The color scale on the top shows the relative expression level of mRNAs: green represents upregulation and red represents downregulation. (**B**) The ratio of protein-coding RNAs and ncRNAs in differentially expressed genes (fold change ≥2, *P* < 0.01) is presented, and the proportion of different ncRNAs is shown in the smaller pie chart. (**C**) Kyoto Encyclopedia of Genes and Genomes (KEGG) pathway analysis of the differentially expressed genes. The x-axis indicates the counts of genes and the y-axis indicates the terms.

To investigate the potential molecular targets of XBP1s, we transfected HEK293 cells with the phosphomimetic mutant XBP1s-S61D or a blank control plasmid. RNA-Seq analysis revealed similar heatmaps of correlation coefficient values across samples, and there was no crossover between the two groups (Supplementary Fig. S5). A total of 96 transcripts were differentially expressed between the overexpressing S61D and control plasmid cells, including 91 upregulated and 5 downregulated transcripts (fold change ≥2, *P* < 0.01). Hierarchical clustering showed systematic variations in the differentially expressed genes between overexpressing S61D and control plasmid cells (Fig. 5A and Supplementary Table S1). The 91 upregulated genes consisted of protein-coding (80%) and non-coding RNAs (ncRNAs) (20%). Most of the ncRNAs were long ncRNAs (13%) or small nucleolar RNAs (5%) (Fig. 5B). To determine the functions and biological processes involved, all of the differentially expressed genes were mapped using the GO database and KEGG pathway. A GO analysis was performed to determine the gene enrichment in biological processes, cellular components, and molecular functions. The dysregulated genes were involved in the regulation of signal transduction, integral component of membranes, and protein binding (Supplementary Fig. S6 and Supplementary Table S2). The KEGG pathway analysis revealed that the differentially expressed genes were related to multiple signaling pathways, including metabolic pathways (Fig. 5C and Supplementary Table S3). Our results indicate that phosphorylated XBP1s serves as a transcription factor involved in the regulation of various target genes, including metabolic protein-coding genes, FosB, and ncRNAs.

## Discussion

Growing evidence from studies on post-mortem human PD brains as well as genetic and toxicological models suggests that ER stress is a common feature of PD and contributes to neurodegeneration^28–30^. In response to ER stress, the folding and degrading capacity of ER is dynamically adjusted by the induction of a complex signal-transduction network, known as the UPR. The IRE1α/XBP1 pathway, a conserved signaling branch of the UPR, plays an important role in various human pathological conditions. Even though several lines of evidence indicate that the IRE1α/XBP1 pathway is involved in the pathogenesis of neurodegenerative disorders^31, 32^, the underlying mechanism of this UPR pathway in PD remains poorly explored. In the present study, we observed that the expressions of XBP1s mRNA, XBP1s protein, and phosphorylated-IRE1α were significantly increased in a cell model of PD. These results confirm that ER stress is present in this PD model and that the IRE1α/XBP1 pathway is markedly activated. However, Holtz and his colleagues fail to observe the processed XBP1 in primary mesencephalic cultures following MPP^+^ (1 μM) treatment^33^. The difference between our results and their findings may be induced by the different concentrations of MPP^+^ treatment and experimental conditions.

Our current work showed that CDK5 is independent with the activation of IRE1α, but is an important regulator of XBP1s nuclear translocation. The phosphorylation of XBP1s at Ser61 by CDK5 greatly enhanced the nuclear translocation of this transcription factor. A recent study has reported that p38MAPK phosphorylated XBP1s and affected its activity^23^. However, we did not detect p38MAPK expression in MPP^+^-treated neurons. Thus, we hypothesize that p38MAPK activity was lower in these differentiated neurons, and thus, could not phosphorylate XBP1s. Notably, the translocation mechanism of phosphorylated XBP1s from the cytoplasm to the nucleus remains unclear. Most proteins with nuclear functions that enter through nuclear pore complexes (NPCs) require additional carrier proteins or transport factors, such as Importin-β (Impβ1), Importin-13 (Imp13), Importin-α (Impα), and Snurportin-1^34, 35^. The conventional nuclear import pathway of proteins transported by Impβ1 involves binding to the nuclear localization signal either directly or through adaptor molecules, such as Impα or Snurportin-1, to form a complex before translocating across the membrane via the nuclear pore^34^. We speculate that XBP1s is recognized by a heterodimer of Impα/β1 or Imp β1 alone, and then translocated via NPCs in PD.

Several lines of evidence support the view that XBP1s, a transcription factor of the UPR, regulates a subset of UPR target genes related to protein folding, ER/Golgi biogenesis, and ER-associated degradation^36^; however, the regulatory mechanisms and functions of XBP1s in the nucleus in PD are poorly understood. To further investigate the function of XBP1s in PD, RNA-Seq was used to analyze the expression of the genes regulated by the phosphomimetic mutant XBP1s-S61D. Interestingly, we detected 96 differentially expressed genes (fold change ≥2, *P* < 0.01), including 91 upregulated genes and 5 downregulated genes. We hypothesized that the downregulation of these 5 genes is indirectly regulated by XBP1s via other molecules or pathways. Most of the upregulated genes were protein-coding genes (80%) mainly involved in intracellular metabolic processes. Previous studies demonstrated that many metabolic disorders, such as obesity, high glucose, insulin resistance, diabetes, hypertension, and inflammation, are crucial environmental risk factors for PD^37^. Thus, XBP1s may participate in the pathogenesis of PD by regulating multiple metabolic pathways.

The FosB gene encodes both FosB and ΔFosB transcription factors, which belong to the activator protein-1 (AP-1) family^38^. ΔFosB is a truncated form of FosB that lacks 101 amino acids at the C-terminus. The alternative splicing of FosB results in a ΔFosB that lacks two degron domains, thereby increasing stability and accumulation and prolonging the effects of ΔFosB at the promoter site^39^. In both rodent and monkey models of PD, the striatal expression of FosB-related proteins is induced by DA-denervating lesions and treatment with D1 receptor agonists^40^. A recent study found that both FosB and ΔFosB expressions were increased in brain regions containing tuberoinfundibular dopamine neurons, and the nuclear and long-term expression of ΔFosB influenced parkin transcription in MPTP-induced acute PD mouse models^41^. Our RNA-Seq analysis revealed that XBP1s upregulates the expression of FosB. Therefore, we speculate that the XBP1s/FosB system is an important regulatory mechanism in the pathogenesis of PD.

In addition to protein-coding genes, many ncRNAs (20%) were differentially expressed, including long ncRNAs (lncRNAs) and small nuclear RNAs (snoRNAs). A growing number of have studies showed that the deregulation of different classes of ncRNAs is involved in various biological processes related to neurogenesis and neurodegeneration^42, 43^. LncRNAs are a class of non-protein coding transcripts longer than 200 nucleotides, which can regulate gene expression at epigenetic, transcriptional, and post-transcriptional levels via a wide array of mechanisms^44^. A present study has confirmed that lncRNA MALAT1 leaded to the increased stability of a-synuclein and its expression in SH-SY5Y cells^45^. Liu *et al*. found that lncRNA HOTAIR promoted PD induced by MPTP through up-regulating the expression of LRRK2^46^. As a transcription factor, XBP1s may be interacted with many lncRNAs involving in the regulation of the pathogenesis of PD.

SnoRNAs are intermediate-sized ncRNAs (60–300 bp) and components of small nucleolar ribonucleoproteins (snoRNPs), which are complexes involved in the regulation of the post-transcriptional modification of ribosomal RNA. The sequences of snoRNAs are responsible for targeting the assembled snoRNPs to a specific target^47, 48^. SnoRNPs are classified as C/D box or H/ACA box based on their conserved secondary structural characteristics. In mammalians, most snoRNAs are encoded within the introns of protein coding or noncoding genes, i.e.,“host-genes”^49^. Therefore, altered snoRNA expression may result from disease processes and changes in the transcriptional activity of hostgenes. A recent study revealed that box C/D RNA SNORD115 negatively regulated the editing and alternative splicing of the serotonin 2C receptor (5htr2c) pre-RNA in the brains of patients with Prader-Willi syndrome^50^. However, the role of snoRNAs in PD is unknown. In our results, four significantly upregulated snoRNAs, including SNORA52, SNORD15A, SNORD134, and SNORD57, were identified using RNA-Seq. We hypothesized that these dysregulated snoRNAs are involved in the regulation of the editing and alternative splicing of certain gene pre-RNAs, and thus, subsequently influence the gene function.

We showed that the IRE1α/XBP1 pathway is activated in a PD model. Moreover, our results revealed that phosphorylation of XBP1s at Ser61 by CDK5 greatly enhances the nuclear translocation of this transcription factor, which subsequently regulates the transcription of metabolic protein-coding genes, FosB and ncRNAs, in a PD model. This study provides important insights into the pathogenesis of PD and is worthy of further investigation in the context of new therapeutic strategies for PD.

## Materials and Methods

### Plasmids and recombinant proteins

The plasmids encoding full-length mouse FLAG-XBP1s was purchased from Addgene. Full-length XBP1s was subcloned into PEGFP-C1 and pGEX-6p1 respectively. The Ser 61 to Ala (SA) or Asp (SD) mutations were performed using the Fast Mutagenesis System (TransGen Biotech). All GST-fusion proteins were expressed in BL21 (DE3) competent cells (TransGen Biotech) and were purified using GS4B glutathione agarose (GE Healthcare, ΜK).

### Cell cultures and transfection

Primary cortical neurons were prepared from an embryonic 18-day Sprague Dawley rat. Cortices from individual embryos were isolated and dissociated with a mild mechanical trituration. Dissociated cells were plated onto 6-well or 24-well culture plates coated with poly-d-lysine and maintained in DMEM (Invitrogen, Carlsbad, CA) with 10% fetal bovine serum (FBS). Four hours after plating the cells, the medium was replaced with serum-free Neurobasal medium supplemented with 2% B-27 supplement, 0.5 mM glutamine, 25M glutamate, penicillin (100 g/ml) and streptomycin (100 g/ml). After 24 hours of plating, cell division inhibitor Ara-C was added to the medium at a final concentration of 10 μM to remove glial cells. Half volume of the medium was replaced every 3 days. The treatments were performed 7 days after plating, with transfection via Lipofectamine 2000 on the day before, if necessary. HEK293 cell line was cultured in Dulbecco’s modified Eagle’s medium (DMEM) with 10% FBS, penicillin (100 g/ml) and streptomycin (100 g/ml) in incubator (5% CO_2_, 37 °C). Cells were plated onto 6-well or 24-well culture plates, and the transfections were performed using Lipofectamine 2000 when the cells reached confluence of 80–90%. All animal experiments were performed according to approved protocols by the Institutional Animal Care and Use Committee of Huazhong University of Science and Technology.

### Peptides and Mass spectrometric analysis

The peptides used in Mass spectrometric analysis were: (i) Scrambled peptide—ARHLLSEPEKT and (ii) a peptide containing XBP1s Ser61 site—RLTHLSPEEKA. All peptides were synthesized from KareBay TM Biochem, Inc. The peptides were ≧95% pure and kept as 100 μM stock solution at −20 °C. Briefly, 2 μg of purified peptides were incubated with active CDK5/p25 kinase *in vitro* at 30 °C for 30 min, and the resulting peptide mixtures were subjected to tandem mass spectrometry analysis. The scrambled and XBP1s peptide were conjugated to myristic acid in its N-terminal, and used as a membrane-penetrating peptides^26^.

### CDK5/p25 *in vitro* kinase assay

The *in vitro* CDK5/p25 kinase assay was performed according to the manufacturer’s instructions (Millipore). Briefly, 2 μg of purified GST wild type XBP1s or S61A mutant GST-XBP1s fusion proteins were incubated with active CDK5/p25 in CDK5 kinase reaction buffer (8 mM MOPS/NaOH, pH7.0, 200 nM EDTA) plus 20 μM ATP. The reaction was incubation at 30 °C for 30 min and stopped with SDS loading buffer boiling for 5 min. The phosphorylation of substrates analysis was analysed by using Phos S/TP antibody or Phos-tag gels.

### Nuclear Protein Extraction

For nuclear protein extraction from cells in 6-well culture plates, cells were removed from dishes by scraping with 100 μl of buffer A (40 mM Tris-HCl, 10 mM NaCl, 1 mM EDTA, 1 mM DTT, protease inhibitors and 1mM PMSF). Following 15 min of incubation on ice, 5 μl of buffer B were added and vortexed for 5s. The cells were centrifuged for 5 min at 14, 000 × *g* at 4 °C, and supernatants were collected to obtain the cytoplasmic fractions. The pellets were resuspended in 30 μl of nuclear lysis buffer (40 mM Tris-HCl, 420 mM NaCl, 10% glycerol, 1 mM EDTA, 1 mM DTT, protease inhibitors and 1mM PMSF). The suspension was incubated on ice for 30 min. During this incubation, lysates were vortexed every 2 min. Finally, cells were centrifuged for 10 min at 14,000 × *g* to obtain nuclear proteins. Nuclear proteins were isolated using a Nuclear and Cytoplasmic Protein Extraction Kit from Beyotime Biotechnology according to the manufacturer’s instructions, with no modifications. The cytoplasmic and nuclear components were then subjected to Western blots analysis.

### Western blots analysis and Phos-tag SDS-PAGE

Total cell lysates or nuclear extracts were separated by SDS–PAGE and transferred to PVDF membranes. The membrane was blocked in Tris-buffered saline (TBS; pH7.4) with 5% milk or BSA for 1 h, followed by incubation with primary antibody in TBST (pH7.4) with 5% blocking reagent at 4 °C overnight. After the incubation, the membrane was washed three times in TBST, followed by incubation with secondary antibody in TBST for 1 h, and washed again. Immunoblots were developed by using chemiluminescence system, and bands were observed using Kodak exposure films. The following antibodies were used for immunoblot analysis: XBP1 (rabbit, 1:1,000), CDK5 (mouse, 1:1,000), p-p38 (rabbit, 1:1,000) and p38 (rabbit, 1:1,000) (Santa Cruz); IRE1a (rabbit, 1:1,000), p-Ser724 IRE1α (rabbit, 1:1,000) and Phos S/TP (rabbit,1:2,000) (Abcam); GST (mouse, 1:10,000, Cell Signaling Technology); tubulin (mouse, 1:5,000) and β-actin (mouse, 1:5,000) (Sigma-Aldrich); HA (rabbit, 1:3,000), GFP (rabbit,1:3,000) and Histone H3 (rabbit, 1:5,000) (Proteintech); Secondary antibodies, goat anti-rabbit IgG and goat anti-mouse IgG (Jackson ImmunoResearch) were used at 1:20,000. The levels of phosphorproteins were separated and detected by Phos-tag SDS-PAGE. Phos-tag SDS-PAGE gels were purchased from Wako Pure Chemical, and were carried out as the same way as western blots, except that: (a) 8% SDS-PAGE containing 25 mM Phos-tag and 50 mM MnCl_2_ was used; (b) gels were soaked in transfer buffer containing 1 mM EDTA for 40 min before transferred to PVDF membrane.

### Quantitative real-time PCR analysis

The total RNA was extracted from neurons using RNAiso (Takara). Total RNA (2 μg) of each sample was reverse-transcribed using ReverTra Ace® qPCR RT Kit (TOYOBO) in a 20 μl volume. The amplification was carried out in a total volume of 25 μl containing 0.5 μl of each primer, 12.5 μl SYBR® Green Realtime PCR Master Mix (TOYOBO), and 1 μl of cDNA, using an CFX96 Touch real-time PCR system, according to the manufacturer’s instructions. β-actin was amplified as a reference standard for rat. Primers used for rat β-actin forward: 5′-CACCCGCGAGTACAACCTTC-3′ and reverse: 5′-CCCATACCCACCATCACACC-3′; for rat XBP1 splicing forward: 5′-TCAGAGGCAGAGTCCAAGGG-3′, reverse: 5′-AGAAAGGGAGGCTGGTAAGG-3′. The quantitative results were calculated using the 2^−△△Ct^ method and then statistically analyzed.

### Cell survival assay

After undergoing various treatments, the primary cultured neurons were seeded in 96-well plates, and pretreated on the 7th day with peptides (0.1 μM, 1 μM, 10 μM, 30 min) and then treated with MPP^+^. At the end of the experiment, the media was removed and the cells were incubated with MTT solution (5 mg/ml) for 4 h. Then the cells were dissolved in 150 μl of dimethyl sulfoxide (DMSO).The absorbance at 570 nm was measured.

### Immunoprecipitation and Immunofluorescence

Samples were collected in lysis buffer (50 mM Tris-HCl (pH7.4), 150 mM NaCl, 1 mM EDTA, 1 mM DTT, and 1% Triton X-100 with protease inhibitor cocktail). Immunoprecipitations were performed through incubation of 1–2 μg antibodies with lysates 4 °C overnight followed by incubation with Protein G Plus/Protein A Agaroses (Calbiochem, Germany) at 4 °C for 2 h. Finally, the Protein G Plus/Protein A Agaroses were boiled in 2× sample buffer for 10 min. The eluents were detected by Western blots.

For the nuclear translocation assays, neurons or HEK293 cells were directly transfected with plasmids with GFP tag for 24 h and then fixed, counterstained with DAPI, and visualized under a fluorescence microscope (Olympus), and at least one hundred cells were counted for each treatment.

### RNA-Seq and data analysis

HEK293 cells were cultured in 6-well plates and transfected with pEGFP-XBP1s-S61D (XBP1s-S61D) and control vector plasmids using Lipofectamine 2000 for 24 hours. Total RNA was isolated using Trizol Reagent (Takara) according to the manufacturer’s protocol. Quantification and quality evaluation were performed by Agilent 2100 Bioanalyzer. For library preparation, poly-A mRNA was extracted from total RNA using OligodT magnetic beads and cleaved to fragments by interrupted buffer. The fragmented RNAs were reverse transcribed into cDNA using random N6 primers, and the cDNAs were selected for PCR amplification as templates. A mixed cDNA sample was prepared and sequenced using the Illumina HiSeq™ 2000. Clean reads were mapped to human reference gene sequences and human reference genome sequences using BWA and Bowtie2. The gene expression level was calculated using RNA-Seq by Expectation Maximization (RSEM) software. Differential expressed genes (DEGs) were identified by absolute value of fold change ≥2 and *P* < 0.01. The data of DEGs were normalized by employing Z-score transformation approach. The expression pattern of DEGs between XBP1s-S61D and control groups was showed by Hierarchical Clustering. Pathway and GO analyses were applied to determine the roles of the DEGs in the regulation of multiple biological processes. GO were performed by the Database for Annotation, Visualization and Integrated Discovery (DAVID, http://david.abcc.ncifcrf.gov), and Kyoto Kyoto Encyclopedia of Genes and Genomes database (KEGG, http://www.genome.ad.jp/kegg/) was used to analyze the potential functions of the target DEGs in pathways.

### Statistical analysis

All of the data were presented as mean ± S.D. Comparisons between groups was determined by unpaired two-tailed Student’s t-tests, where *P* < 0.05 was considered to be statistically significant. All experiments were repeated at least in three independent times.

## Electronic supplementary material

Supplementary information
