# Supplementary material for: CDK5-mediated phosphorylation of XBP1s contributes to its nuclear translocation and activation in MPP+-induced Parkinson’s disease model
Source: Sci Rep. 2017 Jul 17;7:5622. doi: 10.1038/s41598-017-06012-6 (PMC5514026; doi:10.1038/s41598-017-06012-6)
Supplement: Supplementary file 1 — Supplementary information [file 41598_2017_6012_MOESM1_ESM.pdf]

Supplementary information

CDK5-mediated phosphorylation of XBP1s contributes to its nuclear translocation and activation in MPP$^+$-induced Parkinson's disease model

Feng-Juan Jiao[1, 2, 4], Qing-Zhi Wang[1, 2, 3, 4], Pei Zhang[1, 2], Jian-Guo Yan[1, 2], Zheng Zhang [1, 2], Feng He [1, 2], Qian Zhang[1, 2], Ze-Xi Lv [1, 2], Xiang Peng [1, 2], Hong-Wei Cai[1, 2], Bo Tian[1, 2, 5]

[1.] Department of Neurobiology, Tongji Medical School, Huazhong University of Science and Technology, 13 Hangkong Road, Wuhan, Hubei Province, 430030, P. R. China

[2.] Institute for Brain Research, Huazhong University of Science and Technology, 13 Hangkong Road, Wuhan, Hubei Province, 430030, P. R. China

[3.]Medical School, Hubei Ploytecnic University, Huangshi, Hubei Province, P. R. China

[4.] These authors contribute equally to this work.

[5.] Correspondence should be addressed to Bo Tian

Email: tianbo@mails.tjmu.edu.cn

Phone: +86(27)83692630

Fax: +86(27)83692602

Address: 13 Hangkong Road, Wuhan, Hubei Province, 430030, P. R. China

Supplementary Materials and methods

CDK5 RNAi lentivirus and infection

CDK5 and control RNAi lentiviruses were purchased from GeneChem. The two complementary DNA oligonucleotides were chemically synthesized to construct the 19-nucleotide (corresponding to rat CDK5 nt positions 639–657)[1] short hairp in RNA (shRNA) cassettes: 5'-GATCCCCGAGGATCTTTCGACTG-CTATTCAAG AGATAGCAGTCG-AAAGATCCTC-TTTTTGGAAA-3' and 5'-AGCTTTTCCAA AAA-GAGGATCTTTCGACTGCTATCTCTTGAATAGCAGT-CGAAAGATCCTC-G GG-3'. Lentiviruses were applied to primary cultured neurons on the fourth day *in vitro* and treatments were performed 48h after infection.

Bioinformatics analyses

Prediction of CDK5 kinase-specific phosphorylation sites was analyzed with high stringency using both GPS[2] (http://gps.biocuckoo.org) and Scansite[3] (http://scansite.mit.edu/). Scansite can analyze large amounts of protein data by using a web service. The key feature of Scansite is the prediction of motif-relevant sites in a given protein. Scores in Scansite start at 0.000 if the sequence optimally matches the given motif, and the scores increase for sequences as they diverge from the optimal match. If your site ranks in the best 0.2% of all sites, that is quite good (this is the "high stringency" threshold value we use for most of the motifs). Group-based Prediction System (GPS) was a kinase-specific phosphorylation sites predictor. The training data were taken from Phospho.ELM 6.0. And then the theoretically maximal false positive rate (FPR) of each PKs cluster was calculated through a simple approach. The three thresholds of GPS were decided based on calculated FPRs. For serine/threonine kinases, the high, medium and low thresholds were established with FPRs of 2%, 6% and 10%. The score value was calculated by GPS algorithm to evaluate the potential of phosphorylation, and the higher of the value, the more potential the residue is phosphorylated. The cutoff value represented the threshold, and the different threshold means different precision, sensitivity and specificity.

Supplementary Results

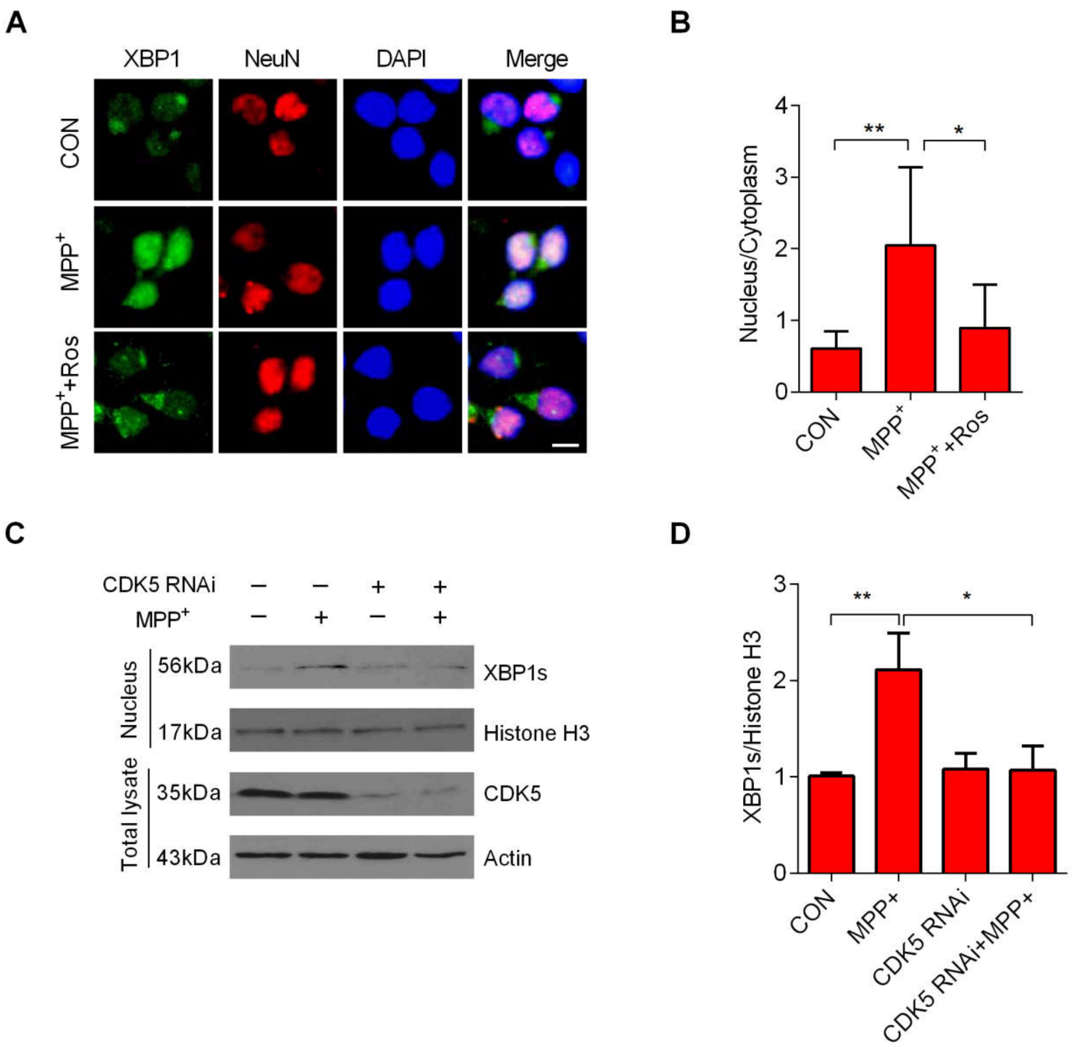

Figure S1. Nuclear translocation of XBP1sinduced by MPP$^+$ is dependent on CDK5. (A) Neurons were pretreated with Ros (10μM, 30 min), and then incubated the neurons with MPP$^+$ (100μM, 12h).Neurons were treated with MPP$^+$ (100μM) for 12h. The location of XBP1 in neurons was detected by immunofluorescence. **(B)** Statistical analysis of the fluorescent intensity of nucleus/cytoplasm in neurons. **(C)** Neurons were infected with control or CDK5RNAi lentivirus for 48 h, then treated with MPP$^+$ (100μM) for 12h. CDK5 levels in whole cell lysates and XBP1s in nuclear fractions were determined by immunoblotting, using Actin and Histone H3 as markers of total lysates and nuclei respectively. **(D)** The relative level of XBP1s protein in the nuclear lysates of CDK5 RNAi lentivirus-infected neurons was quantified by densitometry. Data are mean ± s.d. of n=3 independent experiments. Significance was determined by

unpaired Student's t test. **P*<0.05, ***P*<0.01.

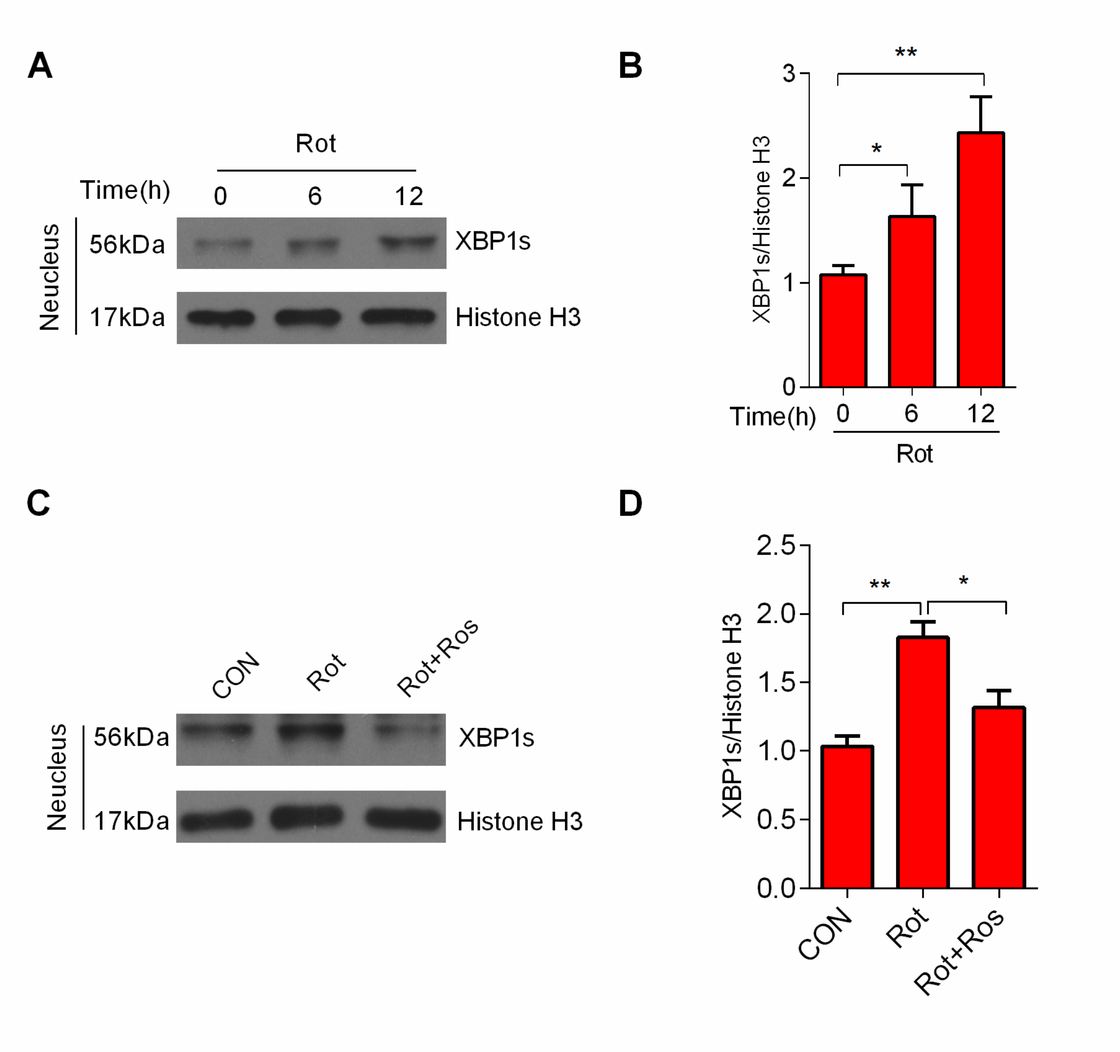

Figure S2. Nuclear translocation of XBP1s occurs in a CDK5-dependent manner in rotenone-induced Parkinson's disease model. (A) A longer treatment with rotenone (Rot) (100nM) induced the upregulation of XBP1s protein in the nuclear extracts of neurons. **(B)** The relative level of XBP1s protein in the nuclear lysates of rotenone-treated neurons was quantified by densitometry. **(C)** Pretreatment with Roscovitine (Ros) (10μM) reduced the rotenone-induced nuclear translocation of XBP1s in neurons. **(D)** The relative level of XBP1s protein in the nuclear lysates of rotenone-treated neurons was quantified by densitometry. Data are mean ± s.d. of n=3 independent experiments. Significance was determined by unpaired Student's t test (B and D). **P*<0.05, ***P*<0.01.

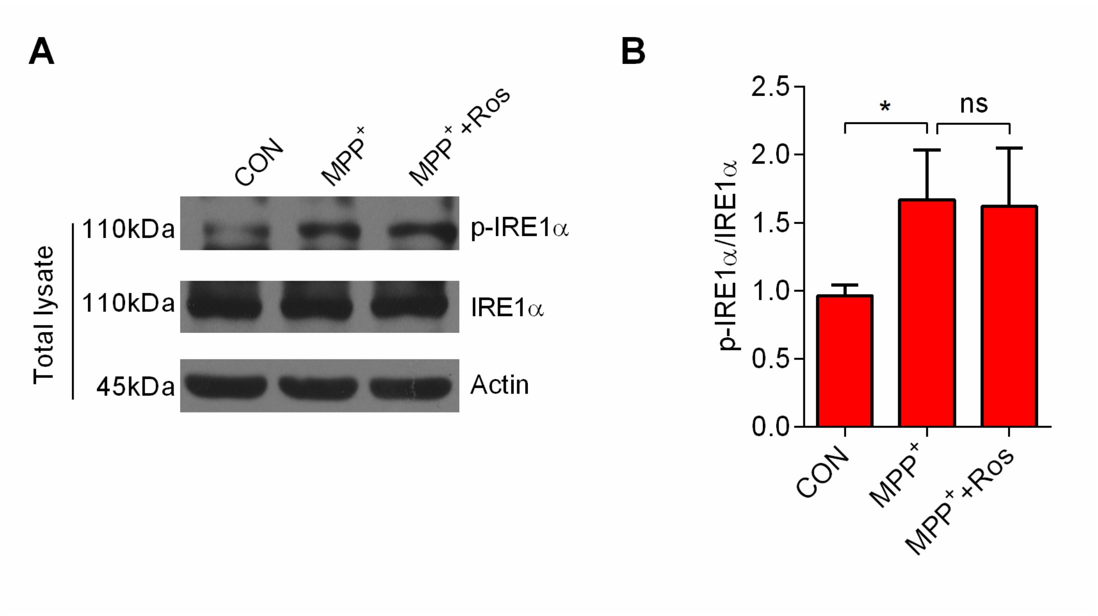

Figure S3. The activation of IRE1α is independent on CDK5 in MPP$^+$-induced Parkinson's disease model. (A) Activation of p-IRE1α was unaffected by Ros (10μM) in MPP$^+$-treated neurons. Primary cultured neurons were pretreated with Roscovitine (Ros) (10μM, 30 min), and then incubated the neurons with MPP$^+$ (100μM, 12h). **(B)** The relative level of p-IRE1α protein in the total lysates of MPP$^+$-treated neurons was quantified by densitometry. Data are mean ± s.d. of n=3 independent experiments. Significance was determined by unpaired Student's t test *P<0.05.

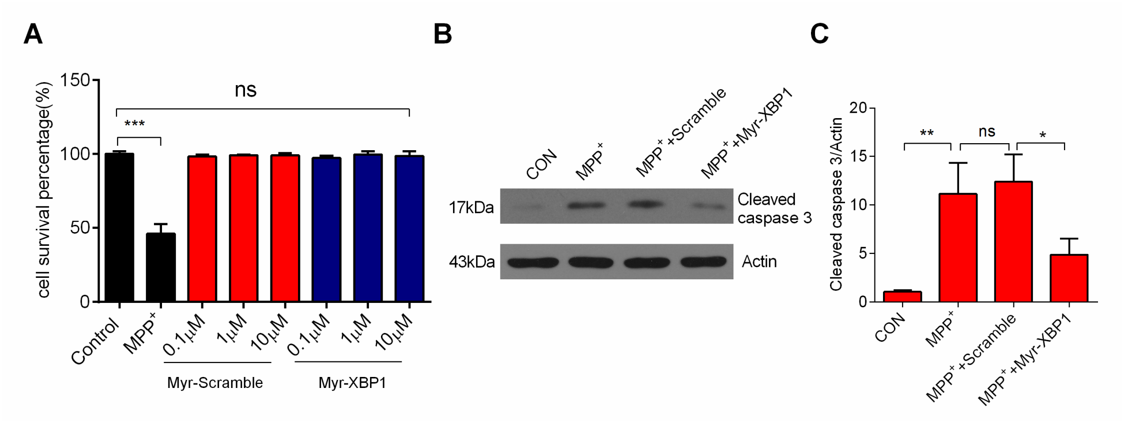

Figure S4. The Myr-XBP1 peptides has no effect on cell survival without MPP$^+$ treatment and has protect effects on MPP$^+$-induced cell apoptosis. (A) Primary cultured neurons were pretreated with Myr-XBP1 peptide or scrambled peptide (0.1μM, 1μM, 10μM, 24h), and then cell survival assay were performed by MTT. **(B, C)** Primary cultured neurons were pretreated with Myr-XBP1 peptide or scrambled

peptide (0.1μM, 30min), and then treated with MPP$^+$ (250μM, 24h). Caspase3 activity was determined by immunoblotting. Data are mean ± s.d. of n=3 independent experiments. Significance was determined by unpaired Student's t test. *P<0.05, **P<0.01, ***P < 0.001.

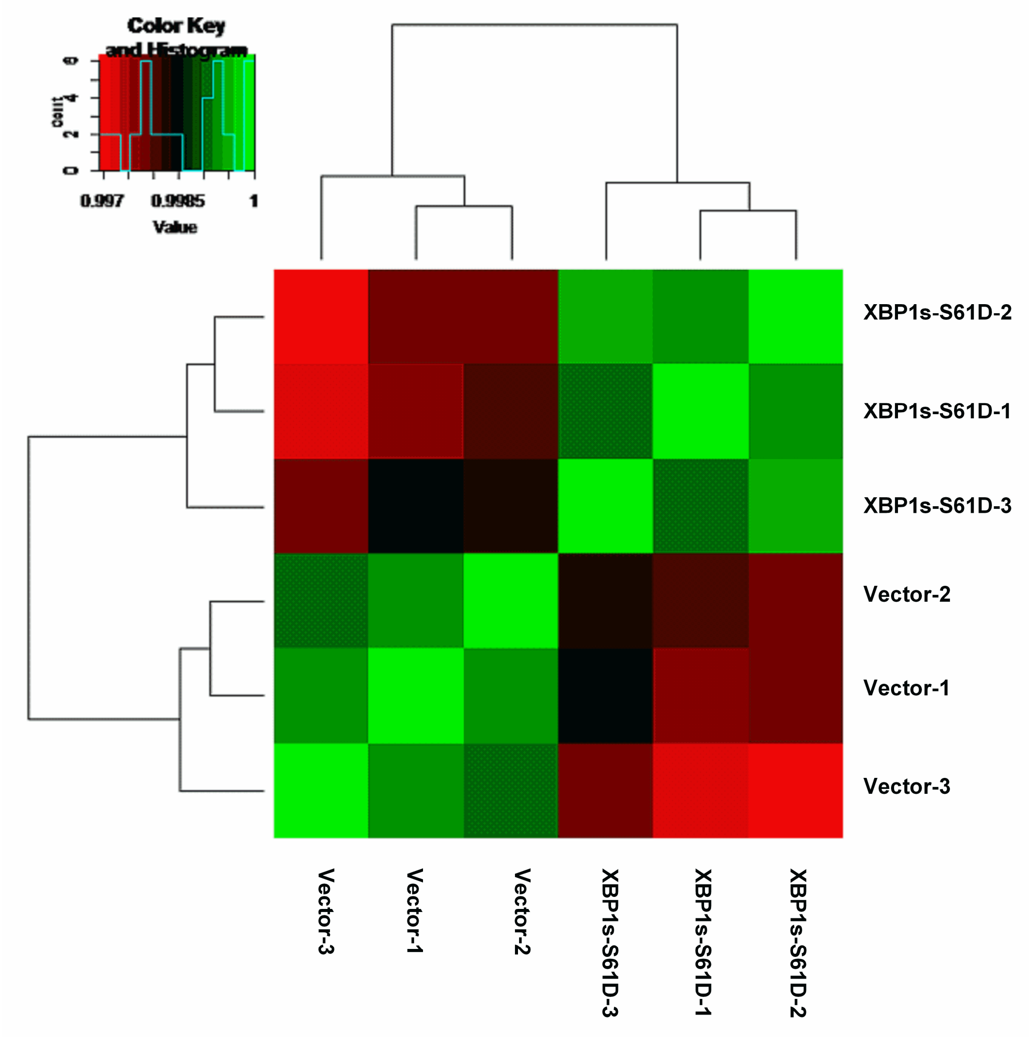

Figure S5. Heatmap of correlation coefficient values acrossing samples. Gradient color barcode at the right top indicates the minimum value in white and the maximum in blue. If one sample is highly similar with another one, the correlation value between them is very close to 1.

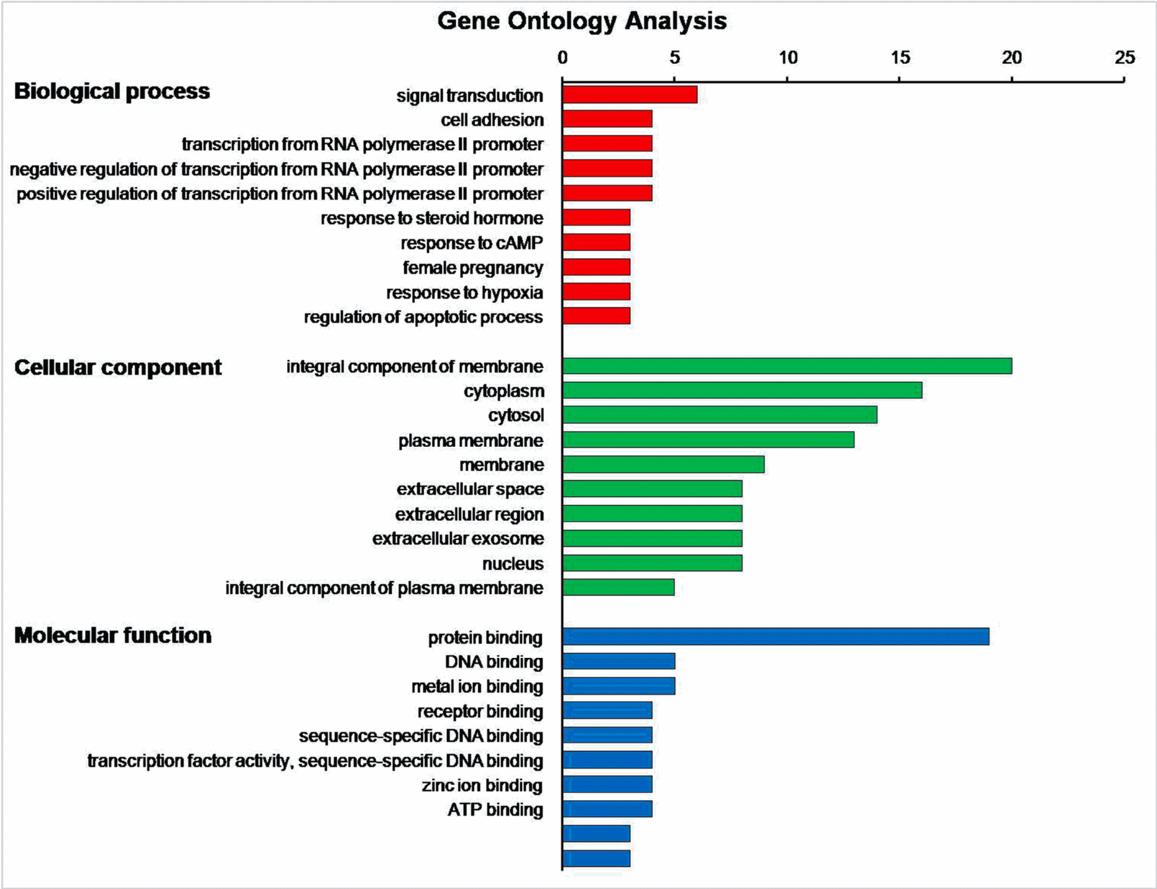

Figure S6. Gene ontology (GO) assignments for the differentially expressed genes. The differentially expressed genes matches GO various ontology categories of biological process, cellular component and molecular function. The x-axis indicates the counts of the genes and the y-axis indicates the GO terms.

Table S1. The differentially expressed genes in XBP1s-S61D overexpressing cells.

Symbol	Vector -1	Vector -2	Vector -3	GFP-XBP1s -1	GFP-XBP1s -2	GFP-XBP1s -3	fold change	p value	Regulation
KLK14	0.31	0.1	0.31	0.96	1.41	1.29	5.08	2.95E-03	up
LOC105369632	0.28	0.55	0.56	2.87	1.75	2.31	4.99	5.35E-03	up
SNORA52	2.08	2.07	2.1	10.83	7.68	10.9	4.71	1.90E-03	up
TTC6	0.03	0.01	0.01	0.09	0.06	0.08	4.60	5.59E-03	up
TMC1	0.1	0.03	0.03	0.25	0.18	0.21	4.00	6.62E-03	up
LOC101927143	0.16	0.3	0.07	0.59	0.77	0.76	4.00	3.96E-03	up
FAM74A4	0.16	0.16	0.17	0.73	0.67	0.44	3.76	7.04E-03	up
LMNTD1	0.16	0.36	0.09	0.77	0.69	0.77	3.66	3.17E-03	up
RASAL1	0.06	0.11	0.03	0.2	0.24	0.26	3.50	4.69E-03	up

FOS	2.68	2.81	2.08	9.26	8.27	8.77	3.47	6.75E-05	up
FOSB	1.57	1.53	1.21	5.28	3.92	5.56	3.42	2.57E-03	up
C9orf173	0.2	0.1	0.3	0.72	0.64	0.69	3.42	1.49E-03	up
LINC00605	0.07	0.07	0.14	0.32	0.36	0.27	3.39	3.08E-03	up
ZNF843	0.05	0.1	0.05	0.21	0.22	0.22	3.25	9.10E-04	up
LOC101927780	0.02	0.02	0.02	0.08	0.06	0.05	3.17	7.97E-03	up
RNF151	0.13	0.13	0.14	0.42	0.42	0.42	3.15	1.10E-07	up
IZUMO4	0.33	0.33	0.33	1.14	0.81	1.14	3.12	3.13E-03	up
RFPL3	0.25	0.31	0.33	1	1.04	0.68	3.06	6.34E-03	up
VN1R1	0.13	0.2	0.13	0.41	0.56	0.42	3.02	4.49E-03	up
TMC4	0.17	0.08	0.21	0.43	0.44	0.48	2.93	2.00E-03	up
RNVU1-6	1.59	0.79	0.8	2.48	3.36	3.34	2.89	7.03E-03	up
SNORD15A	0.91	1.81	0.92	3.79	2.88	3.81	2.88	5.97E-03	up
ANKRD20A3	0.39	0.2	0.19	0.71	0.85	0.68	2.87	4.32E-03	up
SNORD134	2.81	4.2	4.26	10.96	10.36	11.04	2.87	1.73E-04	up
SENP3-EIF4A1	2.2	1.64	4.06	7.48	8.26	6.83	2.86	4.34E-03	up
GAS6-AS1	0.23	0.58	0.25	0.86	1.12	0.98	2.79	9.64E-03	up
INHA	0.29	0.22	0.29	0.75	0.84	0.53	2.65	9.79E-03	up
PTCHD4	0.47	0.7	0.63	1.61	1.9	1.21	2.62	9.98E-03	up
SAPCD1-AS1	1.65	1.98	1.67	3.95	4.35	5.54	2.61	4.35E-03	up
SAMD7	0.13	0.07	0.09	0.24	0.25	0.25	2.55	1.12E-03	up
MUC1	1.27	0.79	0.46	2.18	2.07	2.14	2.54	5.56E-03	up
TMEM86B	1.42	1.3	0.9	3.26	3.24	2.67	2.53	1.76E-03	up
LOC729867	0.2	0.26	0.33	0.62	0.69	0.69	2.53	8.02E-04	up
PNPLA7	0.44	0.51	0.52	1.34	0.99	1.37	2.52	3.96E-03	up
ATHL1	6.59	5.38	6.47	16.65	14.69	14.89	2.51	2.25E-04	up
TNFRSF25	0.82	0.99	1.37	2.74	2.66	2.52	2.49	8.31E-04	up
HDAC10	8.59	8.29	9.27	23.77	20.72	19.5	2.45	6.36E-04	up
DRD4	0.38	0.38	0.54	1.02	1.04	1.11	2.44	4.82E-04	up
SLC6A10P	2.37	1.73	2.19	5.92	5.25	4.13	2.43	5.68E-03	up
AMT	0.71	0.93	1.02	2.21	2.48	1.73	2.41	6.22E-03	up
CORO6	1.76	1.89	2.39	5.19	5.03	4.28	2.40	1.15E-03	up
NOXA1	1.6	1.02	0.82	2.86	2.43	2.96	2.40	4.90E-03	up
MAMDC4	0.78	0.67	0.71	1.8	1.59	1.76	2.38	1.58E-04	up
SIRPG-AS1	0.96	0.53	0.95	2.12	1.61	2.07	2.38	6.52E-03	up
IDUA	0.7	0.79	0.47	1.41	1.47	1.76	2.37	3.44E-03	up
AOC3	0.66	0.64	0.56	1.56	1.22	1.61	2.36	2.61E-03	up
CCDC78	1.42	1.28	1.56	3.15	2.79	4.05	2.35	7.58E-03	up
MEGF6	0.26	0.26	0.24	0.67	0.56	0.54	2.33	1.19E-03	up
MTRNR2L5	1.39	0.67	1.13	2.29	2.48	2.64	2.32	3.83E-03	up
AMH	12.36	12.03	13.93	30.69	30.3	27.64	2.31	1.17E-04	up
LPAR2	0.92	1.03	0.87	1.97	2.3	2.23	2.30	3.81E-04	up
MROH6	0.79	0.88	0.93	2.23	1.7	1.98	2.27	2.24E-03	up
EGR1	16.08	19.12	17.61	45.82	36.07	37.45	2.26	2.20E-03	up

ZNF295-AS1	0.21	0.21	0.21	0.54	0.44	0.44	2.25	1.39E-03	up
GRB7	0.1	0.09	0.15	0.25	0.26	0.25	2.24	1.76E-03	up
KRT42P	0.17	0.21	0.14	0.35	0.44	0.37	2.23	3.29E-03	up
MIR5096	51.99	41.28	59.32	114.37	91.86	133.2	2.22	8.81E-03	up
SCNN1D	1.24	1.42	1.43	3.29	2.77	2.97	2.21	5.47E-04	up
PDIA2	1.62	1.92	1.28	3.19	3.29	4.15	2.21	5.57E-03	up
SAA2	0.52	0.33	0.39	0.86	0.97	0.9	2.20	1.54E-03	up
LOC101928266	0.19	0.23	0.39	0.65	0.58	0.55	2.20	8.90E-03	up
PIP5KL1	0.63	0.46	0.45	1.2	0.9	1.27	2.19	8.78E-03	up
YJEFN3	3.67	4.07	5.22	9.69	10.68	7.97	2.19	5.04E-03	up
RHPN1	3.43	3.42	3.71	8.62	7.66	6.79	2.18	1.48E-03	up
LINC01441	1.63	2.03	1.03	3.22	3.83	3.02	2.15	9.11E-03	up
SSPO	0.07	0.07	0.07	0.15	0.16	0.14	2.14	1.57E-04	up
C2orf66	0.18	0.27	0.18	0.43	0.48	0.43	2.13	2.32E-03	up
NRBP2	2.29	2.25	2.62	5.44	4.9	4.84	2.12	2.82E-04	up
ACTA1	1.06	1.1	1.17	2.24	2.67	2.11	2.11	2.03E-03	up
RHBDL1	4.3	4.26	3.04	7.78	8.23	8.33	2.10	6.84E-04	up
PAQR6	1.05	1.14	1.19	2.48	2.24	2.35	2.09	1.07E-04	up
MAN2C1	12.62	13.29	12.65	27.38	28.08	25.16	2.09	1.02E-04	up
RNASEK-C17orf49	0.48	0.48	0.49	1	1.02	1.01	2.09	1.54E-07	up
COL7A1	1.96	1.87	1.8	3.98	4.16	3.59	2.08	3.10E-04	up
KCND1	0.61	0.63	0.68	1.49	1.28	1.23	2.08	1.09E-03	up
PDXDC2P	7.39	6.63	7.4	14.41	16.37	13.68	2.08	8.04E-04	up
LOC100288152	3.31	3.53	4.76	8.71	7.42	7.86	2.07	2.18E-03	up
PPFIA4	1.44	1.23	1.02	2.79	2.45	2.37	2.06	1.79E-03	up
FANK1	0.15	0.15	0.23	0.31	0.39	0.39	2.06	7.76E-03	up
FOXH1	1.74	1.59	1.95	3.82	3.62	3.4	2.05	3.18E-04	up
FMNL1	0.41	0.46	0.5	0.94	0.98	0.89	2.05	2.00E-04	up
SLC27A3	2.17	2.08	2.27	4.54	4.25	4.53	2.04	3.25E-05	up
KIFC2	6.84	6.07	6.82	14	13.03	13.01	2.03	8.14E-05	up
KCNAB3	1.17	1.13	1.06	2.49	1.99	2.33	2.03	1.59E-03	up
PYGM	0.4	0.31	0.55	0.76	0.9	0.89	2.02	6.68E-03	up
C1orf229	1.11	0.75	0.68	1.7	1.89	1.53	2.02	7.04E-03	up
SNORD57	25.73	25.64	23.12	44.61	57.26	47.91	2.01	2.95E-03	up
RPGRIP1	0.34	0.31	0.31	0.62	0.55	0.76	2.01	6.65E-03	up
WDR90	5	5.27	5.98	11.37	11.06	10.18	2.01	2.92E-04	up
SULT1A4	6.71	5.69	4.17	9.56	12.8	10.83	2.00	9.82E-03	up
CHRD	0.7	0.68	0.35	1.22	1.1	1.14	2.00	8.32E-03	up
IZUMO1	0.64	0.51	0.52	0.2	0.27	0.34	0.49	7.86E-03	down
FAM66D	0.91	0.69	0.72	0.31	0.25	0.27	0.36	2.21E-03	down
SSSCA1-AS1	0.7	0.61	0.8	0.36	0.09	0.18	0.30	6.92E-03	down
PACRG	0.3	0.43	0.32	0.07	0.13	0.1	0.29	4.72E-03	down
HERC2P3	0.53	0.4	0.5	0.09	0.16	0.03	0.20	2.13E-03	down

Table S2. GO analysis of the differentially expressed genes.

Term	Count	Gene Names
**Biological process**		
signal transduction	6	RASAL1,TNFRSF25, GRB7, INHA, RHBDL1, RHPN1
cell adhesion	4	SSPO, AOC3, COL7A1, IZUMO1
transcription from RNA polymerase II promoter	4	FOS, FOSB, EGR1, FOXH1
negative regulation of transcription from RNA	4	FOSB, EGR1, FOXH1, HDAC10
positive regulation of transcription from RNA polymerase II promoter	4	FOS, FOSB, EGR1, FOXH1
response to steroid hormone	3	ACTA1, DRD4, PAQR6
response to cAMP	3	FOS, FOSB, PYGM
female pregnancy	3	FOS, FOSB, MUC1
response to hypoxia	3	EGR1, MUC1, PYGM
regulation of apoptotic process	3	TNFRSF25, EGR1, INHA
**Cellular component**		
integral component of membrane	19	MAMDC4, PACRG, RNASEK-C17orf49, AOC3, FMNL1, IZUMO1, LPAR2, MUC1, PNPLA7, PTCHD4, KCNAB3, KCND1, PAQR6, RHBDL1, SCNN1D, SLC27A3, TMC1, TMC4, TMEM86B
cytoplasm	16	MTRNR2L5, AOC3, EGR1, FANK1, HDAC10, INHA, KIFC2, LMNTD1, MUC1, NRBP2, PIP5KL1, PYGM, KCNAB3, RNF151, SULT1A4, TMEM86B
cytosol	14	FOS, NOXA1, PACRG, PPFIA4, RASAL1, TNFRSF25, ACTA1, FMNL1, GRB7, MAN2C1, NRBP2, PYGM, RHPN1, SULT1A4
plasma membrane	13	RASAL1, TNFRSF25, AOC3, DRD4, FMNL1, GRB7, IZUMO1, LPAR2, KCNAB3, KCND1, PAQR6, SCNN1D, VN1R1
membrane	9	FOS, MAMDC4, DRD4, FMNL1, PIP5KL1, RHBDL1, SCNN1D, SLC27A3, TMEM86B
extracellular space	8	SSPO, ACTA1, AMH, CHRD, COL7A1, KLK14, MUC1, SAA2
extracellular region	8	IZUMO4, MTRNR2L5, TNFRSF25, AMH, C2orf66, COL7A1, INHA, MEGF6
extracellular exosome	8	ACTA1, FMNL1, IDUA, KLK14, MUC1, PYGM, SAA2, TMC4
nucleus	8	FOS, FOSB, IZUMO4, PACRG, EGR1, FOXH1, HDAC10, RNF151
integral component of plasma membrane	5	TNFRSF25, DRD4, LPAR2, MUC1, RHBDL1
**Molecular function**		

protein binding	19	FOS, NOXA1, PPFIA4, ACTA1, AOC3, CHRD, COL7A1, DRD4, EGR1, FAM74A4, FOXH1, GRB7, HDAC10, INHA, IZUMO1, LPAR2, MUC1, PYGM, RPGRIP1
DNA binding	5	FOS, FOSB, RNASEK-C17orf49, EGR1, FOXH1
metal ion binding	5	RASAL1, EGR1, HERC2P3, KCND1, ZNF843
receptor binding	4	AMH, IDUA, INHA, IZUMO1
sequence-specific DNA binding	4	FOS, FOSB, EGR1, FOXH1
transcription factor activity, sequence-specific DNA binding	4	FOS, FOSB, EGR1, FOXH1
zinc ion binding	4	EGR1, MAN2C1, RFPL3, RNF151
ATP binding	4	ACTA1, KIFC2, NRBP2, PIP5KL1
transcriptional activator activity, RNA polymerase II core promoter proxi malregion sequence-specific binding	3	FOS, FOSB, EGR1
RNA polymerase II core promoter proxi mal region sequence-specific DNA binding	3	FOS, FOSB, MUC1

Table S3. KEGG analysis of the differentially expressed genes.

Term	Count	Gene Names
Metabolic pathways	5	AOC3, AMT, IDUA, PIP5KL1, PYGM
Glycine, serine and threonine metabolism	2	AOC3, AMT
Amphetamine addiction	2	FOS, FOSB
TGF-beta signaling pathway	2	AMH, CHRD
Insulin resistance	2	PYGM, SLC27A3
Dopaminergic synapse	2	FOS, DRD4
Osteoclast differentiation	2	FOS, FOSB
Alcoholism	2	FOSB, HDAC10
cAMP signaling pathway	2	FOS, AMH
Cytokine-cytokine receptor interaction	2	TNFRSF25, AMH
HTLV-I infection	2	FOS, EGR1
Neuroactive ligand-receptor interaction	2	DRD4, LPAR2
Pathways in cancer	2	FOS, LPAR2

Supplementary Reference

1 Meuer, K. *et al.* Cyclin-dependent kinase 5 is an upstream regulator of mitochondrial fission during neuronal apoptosis. *Cell death and differentiation***14**, 651-661, doi:10.1038/sj.cdd.4402087 (2007).

2 Xue, Y. *et al.* GPS 2.1: enhanced prediction of kinase-specific phosphorylation sites with an algorithm of motif length selection. *Protein Eng Des Sel***24**, 255-260,

doi:10.1093/protein/gzq094 (2011).

3 Obenauer, J. C., Cantley, L. C. & Yaffe, M. B. Scansite 2.0: Proteome-wide prediction of cell signaling interactions using short sequence motifs. *Nucleic acids research* **31**, 3635-3641 (2003).

Figure1B

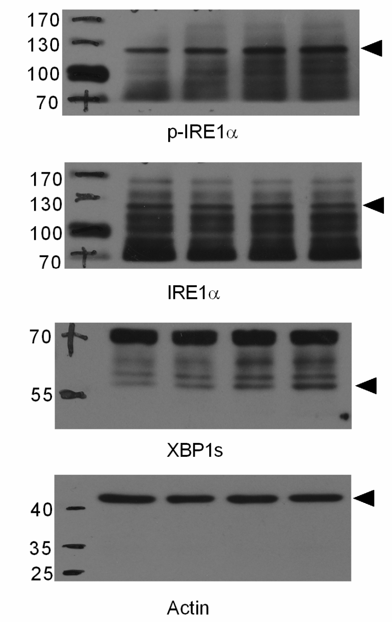

Figure1F

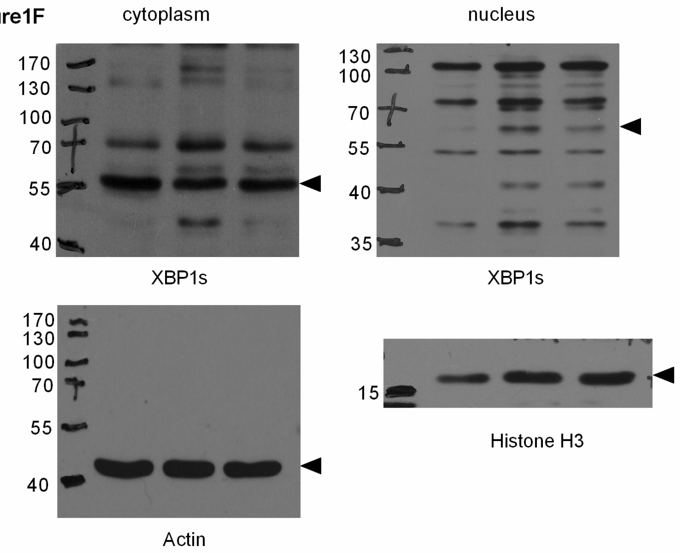

Figure1I

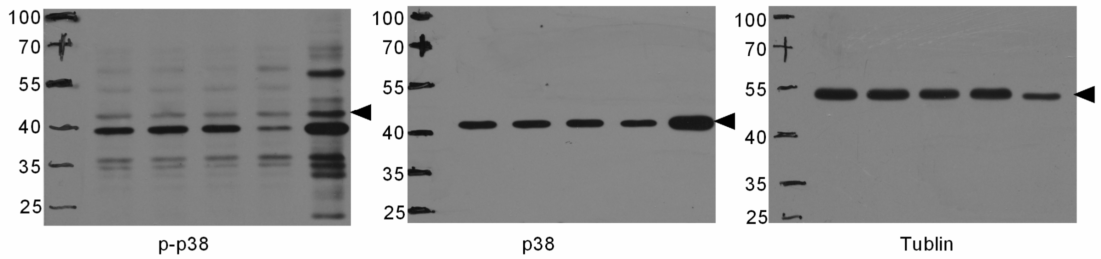

Figure2A

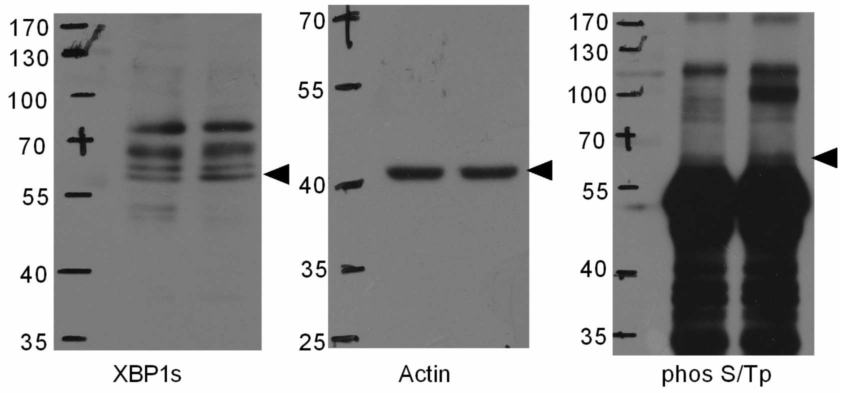

Figure2D

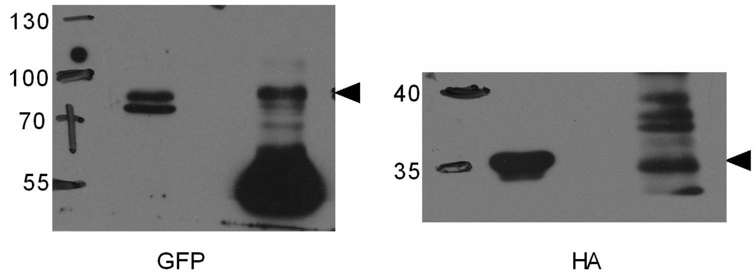

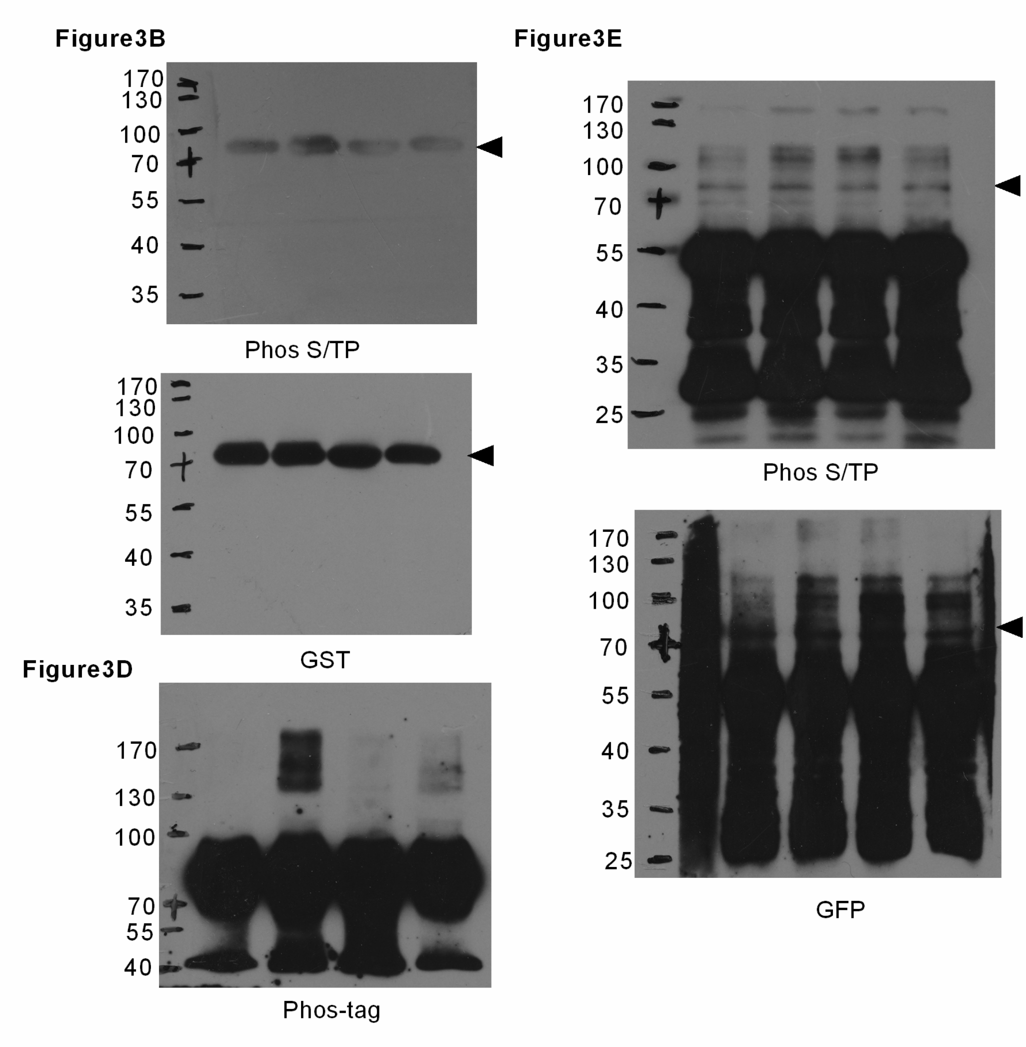
Figure3B
170
130
100
70
55
40
35
Phos S/TP
170
130
100
70
55
40
35
GST
Figure3D
170
130
100
70
55
40
Phos-tag
Figure3E
170
130
100
70
55
40
35
25
Phos S/TP
170
130
100
70
55
40
35
25
GFP

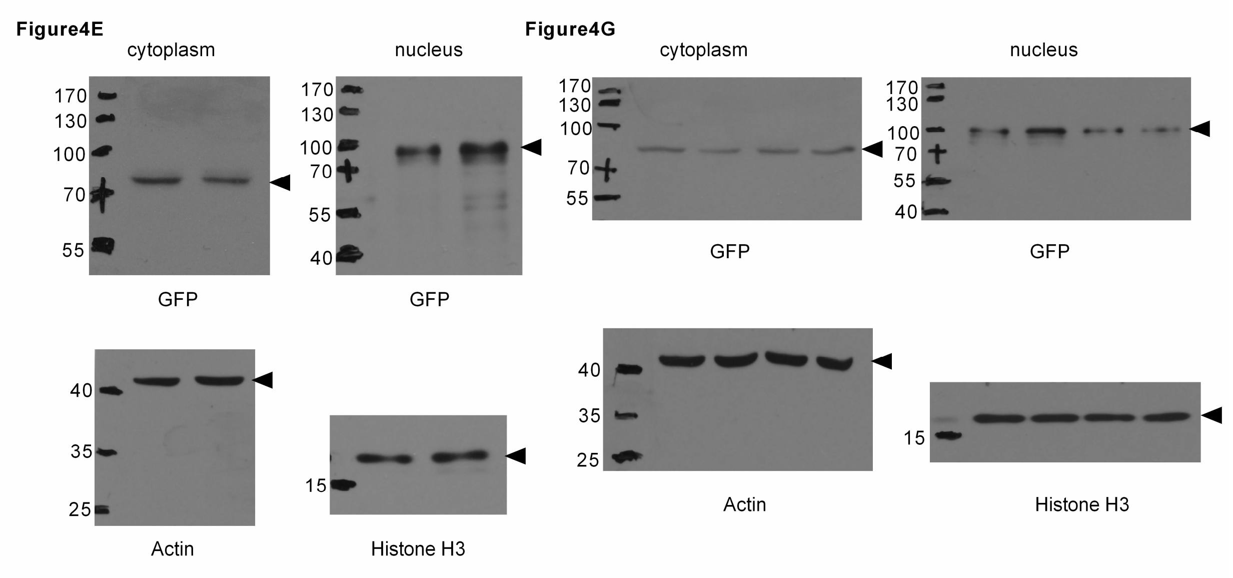
Figure4E
cytoplasm
170
130
100
70
55
GFP
nucleus
170
130
100
70
55
40
GFP
40
35
25
Actin
15
Histone H3
Figure4G
cytoplasm
170
130
100
70
55
GFP
nucleus
170
130
100
70
55
40
GFP
40
35
25
Actin
15
Histone H3
